# Gene network downstream plant stress response modulated by peroxisomal H_2_O_2_

**DOI:** 10.3389/fpls.2022.930721

**Published:** 2022-08-23

**Authors:** Laura C. Terrón-Camero, M. Ángeles Peláez-Vico, A. Rodríguez-González, Coral del Val, Luisa M. Sandalio, María C. Romero-Puertas

**Affiliations:** ^1^Department of Biochemistry and Molecular and Cellular Biology of Plants, Estación Experimental del Zaidín (EEZ), Consejo Superior de Investigaciones Científicas (CSIC), Granada, Spain; ^2^Department of Artificial Intelligence, University of Granada, Granada, Spain; ^3^Andalusian Data Science and Computational Intelligence (DaSCI) Research Institute, University of Granada, Granada, Spain

**Keywords:** abiotic stress, jasmonic acid, peroxisomes, reactive oxygen species, signaling

## Abstract

Reactive oxygen species (ROS) act as secondary messengers that can be sensed by specific redox-sensitive proteins responsible for the activation of signal transduction culminating in altered gene expression. The subcellular site, in which modifications in the ROS/oxidation state occur, can also act as a specific cellular redox network signal. The chemical identity of ROS and their subcellular origin is actually a specific imprint on the transcriptome response. In recent years, a number of transcriptomic studies related to altered ROS metabolism in plant peroxisomes have been carried out. In this study, we conducted a meta-analysis of these transcriptomic findings to identify common transcriptional footprints for plant peroxisomal-dependent signaling at early and later time points. These footprints highlight the regulation of various metabolic pathways and gene families, which are also found in plant responses to several abiotic stresses. Major peroxisomal-dependent genes are associated with protein and endoplasmic reticulum (ER) protection at later stages of stress while, at earlier stages, these genes are related to hormone biosynthesis and signaling regulation. Furthermore, in silico analyses allowed us to assign human orthologs to some of the peroxisomal-dependent proteins, which are mainly associated with different cancer pathologies. Peroxisomal footprints provide a valuable resource for assessing and supporting key peroxisomal functions in cellular metabolism under control and stress conditions across species.

## Introduction

Eukaryotic cells evolve to a stage of organelle compartmentalization in order to increase the efficiency of metabolic processes and to protect cellular components from free radicals such as reactive oxygen and nitrogen species (ROS/RNS), which, in excessive amounts, may be harmful (Gabaldón, 2018). Peroxisomes, which are small organelles found in most eukaryotes, are bounded by a single lipid membrane and have a close relationship with other organelles such as chloroplasts and mitochondria (Kao et al., 2018; Sandalio et al., 2021). Initially, these organelles were regarded as a H_2_O_2_ sink produced from different sources both inside and outside peroxisomes and degraded by catalases and other ROS-inactivating enzymes (Sandalio and Romero-Puertas, 2015; Cross et al., 2016). However, in recent years, biochemical, transcriptomic and proteomic techniques have demonstrated that these organelles are much more complex and perform functions hitherto unknown (Reumann and Bartel, 2016; Sandalio et al., 2021). In fact, the metabolic diversity and plasticity of peroxisomes are remarkable, and new unexpected functions of plant peroxisomes continue to be discovered (Reumann and Bartel, 2016). Key metabolic pathways, such as β-oxidation and photorespiration, are hosted in plant peroxisomes, with the latter pathway being shared with the chloroplast and mitochondria. In addition, the biosynthesis of the phytohormones jasmonic acid (JA), auxin indole-3-acetic acid (IAA) and salicylic acid (SA), together with ROS/RNS metabolism, makes peroxisomes a source of signaling molecules which are essential for the regulation of development processes and plant responses to stress (Sandalio et al., 2021).

Although the identification of other organelle/compartment-dependent signaling communication with the nucleus, termed retrograde signaling, which plays a key role in cell responses to environmental cues, organelle assembly and metabolism, has improved, research into peroxisome-dependent retrograde signaling is in its infancy. Peroxisomes, as sources of signaling molecules, play a key role in triggering adjustments in the transcriptome, which are essential in plant responses to environmental cues. The chemical properties of the ROS hydrogen peroxide (H_2_O_2_) such as stability and diffusibility make this molecule easier to monitor and analyze than other ROS. The specificity of H_2_O_2_-dependent signaling produced in different organelles has previously been demonstrated, particularly in relation to H_2_O_2_ produced in peroxisomes induced either by chemical treatment (Gechev et al., 2005) or H_2_O_2_ disturbances in different mutants (Takahashi et al., 1997; Queval et al., 2007; Chaouch et al., 2010; Sewelam et al., 2014; Su et al., 2018; Romero-Puertas et al., 2022). Thus, transcriptional changes have been analyzed in mutants affected by catalase (CAT), one of the main peroxisomal antioxidants, under different stress conditions such as high or continuous light, CO_2_ shifts and photorespiratory stress (Vanderauwera et al., 2005; Mhamdi et al., 2010; Queval et al., 2012; Sewelam et al., 2014; Kerchev et al., 2016; Waszczak et al., 2016). The transcriptome of the triple mutant cat1 cat2 cat3, showing redox disorders under control conditions, has also been analyzed (Su et al., 2018). Excess H_2_O_2_ in this triple mutant affects genes involved in growth regulation, plant responses to stress and MAPK cascades (Su et al., 2018). The transcriptomes of other mutants affected in glycolate oxidase (GOX), one of the main H_2_O_2_-producing enzymes, involved in peroxisome-located photorespiration, have been analyzed under control and stress situations (Kerchev et al., 2016). All these analyses point to the capacity of peroxisome-produced H_2_O_2_ to directly and/or indirectly modify gene expression. The structure of gene networks and the identification of downstream responses induced by peroxisomal H_2_O_2_ are not well known. In fact, the principal outstanding questions in peroxisome research concern the role of peroxisome-derived ROS and how environmental signals and internal metabolic states of the organelle are translated at the molecular level (Sandalio and Romero-Puertas, 2015; Cross et al., 2016; Rodríguez-Serrano et al., 2016; Kao et al., 2018).

Meta-analysis of different transcriptomes offers a straightforward method to identify common and specific groups of transcriptomic changes. Previous meta-analyses have shown specific signatures for different sources of ROS (Rosenwasser et al., 2013; Willems et al., 2016). However, robust marker transcripts related to peroxisome-dependent signaling are rarely used. Analysis of peroxisomal ROS-dependent transcripts generated in different laboratories would help to obtain marker genes for this organelle-dependent signaling. In this study, we examine both in-house and publicly available data sets derived from the profiling of Arabidopsis gene expression in mutants, as well as treatments with altered peroxisome-dependent ROS, in order to identify a data set of common and specific genes regulated by peroxisomal ROS under different conditions. Furthermore, a search for human orthologs to plant peroxisomal-dependent proteins showed key human proteins mainly involved in the development of different cancers. This analysis should enable us to gain a deeper understanding of the role played by peroxisomes as stress sensors and regulators of cellular responses to adverse conditions resulting in higher resistance.

## Materials and methods

### Plant material and growth conditions

*Arabidopsis thaliana* ecotype Columbia-0 (Col-0) constitutes the genetic background for all plants used in this study. WT, *cat2-2* (Queval et al., 2007), *gox1-1* and *gox2-1* (Rojas et al., 2012) seeds were surface disinfected and stratified for 24–48 h (h) at 4°C and then sown on Murashige and Skoog (MS) 0.5x solid medium (Murashige and Skoog, 1962) and grown at 22°C in 16 h light and 8 h darkness for 14 days (d). Plants were then transferred to petri dishes containing 0.5x liquid MS medium and grown for 24 h. To study the effect of cadmium (Cd), salt (NaCl) or paraquat (PQ) on gene expression, the solution was supplemented with 100 μM CdCl_2_, 100 mM NaCl or 0.5 μM PQ, respectively. For heat shock stress (HS), plants were grown as described above for 14 days and then subjected to 33°C. Seedlings were then collected after 1 and 3 h of treatment and analyzed by qRT-PCR. Microarray samples were taken after 1 and 24 h of Cd treatment.

### RNA isolation from seedlings

Total RNA was isolated from seedlings by the acid guanidine thiocyanate-phenol-chloroform method (Chomczynski and Sacchi, 1987), using the TRIzol reagent ® (MRC) according to the manufacturer’s instructions. RNA was reverse transcribed with the aid of the PrimeScript RT Master Mix (Takara) according to the distributor’s instructions.

### RT-PCR analysis of gene expression

Each 20 μl reaction contained either 1 μl cDNA or a dilution, 200 nM of each primer, and 1x TB Green Premix Ex Taq (Takara). Quantitative real-time PCR was performed on an iCycler iQ5 (Bio-Rad). The samples were initially denatured by heating at 95°C for 3 min followed by 35-cycle amplification and a quantification program (95°C for 30 s, 50–58°C for 30 s, and 72°C for 45 s). A melting curve was conducted to ensure amplification of a single product. The amplification efficiency of primers was calculated using the formula E = [10 (1/a) -1] x 100, where a is the slope of the standard curve. Relative expression of genes was normalized using TUB4 which was selected for normalization by the GrayNorm algorithm (Remans et al., 2014) from five candidate reference genes as described previously in Terrón-Camero et al. (2020). Results were calculated with the ratio according to the Pfaffl method (Pfaffl, 2001). The primers used are described in Supplementary Table S1.

### Microarray data

**Figure 1:**
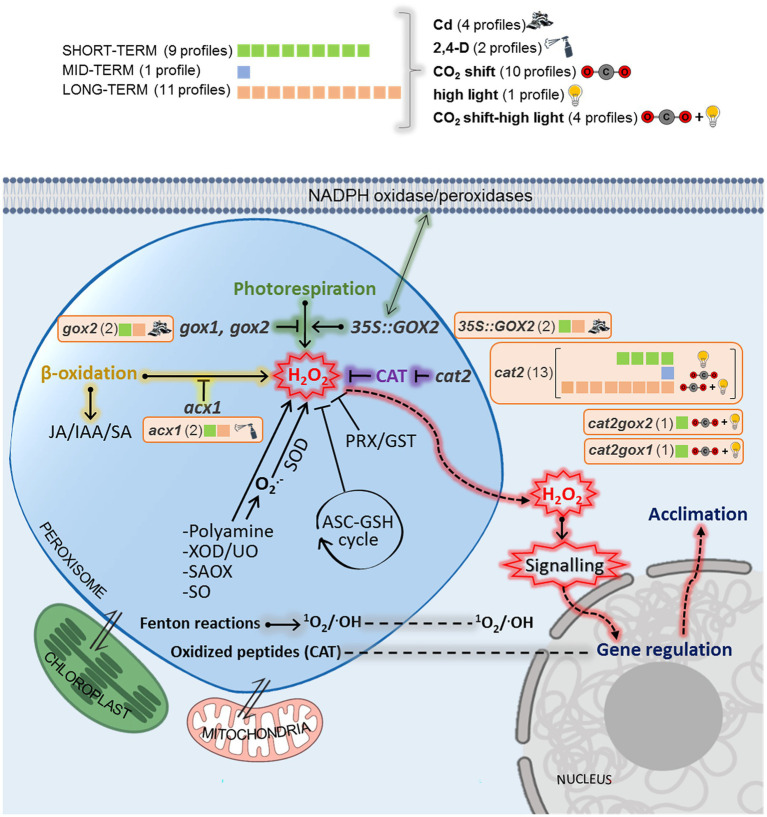
Peroxisomal transcriptional profiles perturbation categories and timing. Transcriptional profiles monitoring peroxisomal H_2_O_2_ perturbations were classified according to the genetic backgrounds used (in italics), chemical treatments/environmental stresses (in bold) and timing used (capital letters) which are detailed in Supplementary Table S2. For each perturbation category, the number of transcriptional profiles is given. *35S::GOX2*: mutants with glycolate oxidase 2 overexpression; *gox1/2:* mutants affected in glycolate oxidase 1/2; *acx1:* mutants affected in acyl Co-A oxidase1; *cat2-2*: mutants affected in catalase 2.

In total, twenty-one lists of genes (profiles) from seven independent studies, showing disrupted peroxisomal H_2_O_2_ metabolism, were collected from the Gene Expression Omnibus repository,^1^ from published data (Figure 1; Supplementary Table S2) and from one transcriptome conducted and analyzed in-house and carried out by the Functional Genomics Lab at the University of Verona (GSE199325; Figure 1; Supplementary Table S2).^2^

### Data processing, cross comparison and data analysis

Differentially expressed genes (DEGs) with respect to their control provided by authors were organized in a database according to treatment, timing and mutant (blue color in gene selection column in Supplementary Table S2). Different groups were compared using Venny 2.0^3^ and Bioinformatics and evolutionary genomics^4^ software to obtain common and specific genes in the different groups (blue color in Venn diagram comparison in gene selection column in Supplementary Table S2). In order to compare all sets of DEGs, we did not take into account the level of expression, but rather its increase or decrease with regard to each respective control condition (Supplementary Table S2). Among the 11 short-time profiles (1–3 h), we selected DEGs from at least five stressed peroxisome profiles and, in the case of nine long-time profiles (≥24 h), we selected DEGs from at least four stressed peroxisome profiles. Classification into different Gene Ontology (GO) categories and functional class enrichment of DEGs of interest were analyzed using the Classification Super Viewer tool,^5^ StringDB,^6^ GeneMania,^7^ Mapman,^8^ and KEGG^9^ using the background for *A. thaliana* and running on default parameters. The PlantGSEA tool^10^ (Yi et al., 2013) was used to perform functional enrichment of the 101 and 86 genes from short and long times, respectively. The analysis was carried out using the Plant Gene Ontology Arabidopsis Information Resource (TAIR) database, comparing our dataset with the complete Arabidopsis genome. The statistical test employed was Yekutieli (FDR < 0.05). Graphs of the most significant GO terms have also been made focusing on biological processes and cellular components using R programming.

### Statistical analysis

Mean values for the quantitative experiments described above were obtained from at least three independent experiments, with no less than three independent samples per experiment. Statistical analyses were performed using a one-way ANOVA test followed by a Student’s *t*-test (value of *p* <0.05). The analyses were carried out with the aid of IBM SPPS Statistics 24. Error bars representing standard error (SEM) are shown in the figures.

### Identification of human orthologs of plant peroxisome-dependent signaling genes

The protein sequences of identified plant peroxisome-dependent signaling genes were extracted from the *A. thaliana* genome version TAIR10, Assembly GCA_000001735.1, using the Ensembl API (Howe et al., 2020). Sequences in FASTA format were used to query the database Ortho DB (Kriventseva et al., 2018) using their API. This database provides evolutionary annotations including rates of ortholog sequence divergence, copy-number profiles, sibling groups and gene architectures. This information enables the creation of groups of orthologs descended from a single gene of the last common ancestor (LCA) of each clade of species identifying *A. thaliana* conserved genes in the human genome.

### Network analysis of human orthologs

We were able to assign human orthologs to 21 of the *A. thaliana* peroxisome-dependent signaling proteins. Network analyses was carried out using Genemania (Warde-Farley et al., 2010) and NetworkAnalyst 3.0 (Zhou et al., 2019). The resulting networks were subjected to enrichment analysis using GO annotation and pathway databases with the aid of NetworkAnalyst.

## Results

### Peroxisomal ROS-perturbed transcriptomic data sets

We compiled data from seven independent publicly available transcriptomic results with different experimental setups related to mutants with altered peroxisomal H_2_O_2_ metabolism in *A. thaliana* (Figure 1; Supplementary Table S2). Genetic modifications involve peroxisomal antioxidant enzymes such as catalase (CAT; *cat2-2* mutants) and peroxisomal ROS-producing enzymes such as glycolate oxidase (GOX; *gox1*, *gox2* mutants) and Acyl-CoA oxidase (ACX; *acx1* mutants). Two double mutants from the genotypes above were also included (*cat2-2* *gox1* and *cat2-2* *gox2*). All transcriptomic analyses were organized in a profile database according to treatment, timing and mutant, resulting in 21 datasheets (Figure 1). We found five categories according to the stress applied: Cd (four profiles), 2,4-D (two profiles), CO_2_ shift (ten profiles), high light (one profile), and combined CO_2_ and high light (four profiles) as described in published studies (Supplementary Table S2). Six genetic backgrounds were distributed in different profiles: *gox2* (two profiles); *35S::GOX2* (two profiles); *cat2* (13 profiles); *cat2gox1* (one profile); *cat2gox2* (one profile) and *acx1* (two profiles). To identify early and late peroxisome-dependent genes, we divided the meta-analysis into three categories depending on the timing of the treatment analyzed: over the short term (0.5–3 h, nine profiles); long term (1–4 days, 11 profiles) and medium term (3–23 h, one profile).

### Identification of peroxisomal ROS-dependent transcriptional changes

**Figure 2:**
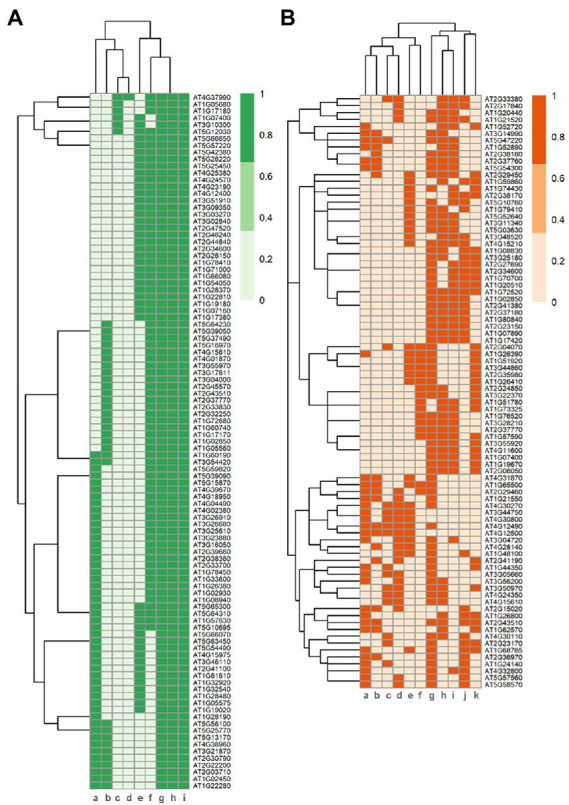
Peroxisomal-ROS-dependent transcriptional changes. Heatmap of DEGs selected from transcriptional profiles related to peroxisome-dependent signaling over the short term **(A)** and long term **(B)**. 1 denotes presence of a gene in a specific profile and 0 no presence. In **(A)**, genes selected are present in at least 5 different profiles and in (B) at least in four. X axis profiles code in **(A)**: (a) CAT2-dependent genes under high light, 180 min; (b) ACX1-dependent genes in plant response to 2,4-D, 1 h; (c) 35S::GOX2-dependent genes in plant response to Cd, 30 min; (d) GOX2-dependent genes in plant response to Cd, 30 min; (e) CAT2-dependent genes, under CO_2_-shift and high light, 180 min; (f) CAT2-dependent genes in a gox1 background, under CO_2_-shift and high light, 180 min; (g) CAT2-dependent genes in a *gox2* background, under CO_2_-shift and high light, 180 min; (h) *cat2* vs. *cat2 gox1*; (i) *cat2 vs. cat2 gox2*. X axis profiles code in **(B)**: (a) ACX1-dependent genes in plant response to 2,4-D, 72 h; (b) 35S::GOX2-dependent genes in plant response to Cd, 24 h; (c) GOX2-dependent genes in plant response to Cd, 24 h; (d) CAT2-dependent genes after CO_2_-shift, 4 days, under short-day conditions; (e) CAT2-dependent genes under CO_2_ shift, under long-day conditions, 4 days; (f) CAT2-dependent genes after CO_2_-shift, 2 days, under short-day conditions; (g) CAT2-dependent genes under CO_2_ shift, under short-day conditions, 4 days; (h) CAT2-dependent genes after CO_2_-shift, 2 days, under long-day conditions; (i) CAT2-dependent genes after CO_2_-shift, 4 days, under short-day conditions; (j) CAT2-dependent genes, under CO_2_-shift and high light, 24 h; (k) CAT2-dependent genes under CO_2_ shift, 24 h.

Differentially expressed genes (DEGs) were defined in each datasheet, resulting in a number ranging from 6,266 to only four (Supplementary Table S3). Interestingly, the lowest number of DEGs was observed in comparisons *cat2 vs.* *cat2gox2* and *cat2 vs.* *cat2gox1*, as showed in a previous study (Kerchev et al., 2016). A large majority of the datasheets (18 out of 21) had more than a 100 DEGs (Supplementary Table S3). After short-term profile comparisons, we selected DEGs present in a minimum of five transcriptional profiles (55% of the profiles analyzed) originating from at least four independent studies. These conditions fit with our objective to find common footprints for peroxisome-dependent signaling from different origins. For long-term profile comparisons, however, we selected DEGs present in a minimum of four transcriptional profiles and found a small number of DEGs common to five profiles, probably due to the side effects of different stresses applied over a longer period of time, which may interfere with the ability to obtain common genes due to persistent stress situations. On the basis of these criteria, we found that 101 genes (about 1% of the 9,452 DEGs analyzed) were commonly regulated during short-time treatments (Figure 2A; Supplementary Table S4) and 86 genes (about 1% of the 8,620 DEGs analyzed) during long-time treatments (Figure 2B; Supplementary Table S5). Only six genes were common to both the short- and long-term genes selected (in bold in Supplementary Table S4). As we found only one profile for medium-term treatments, no further analyses were carried out for this period.

### Early peroxisome-dependent transcriptional regulation of pathways and gene families

**Figure 3:**
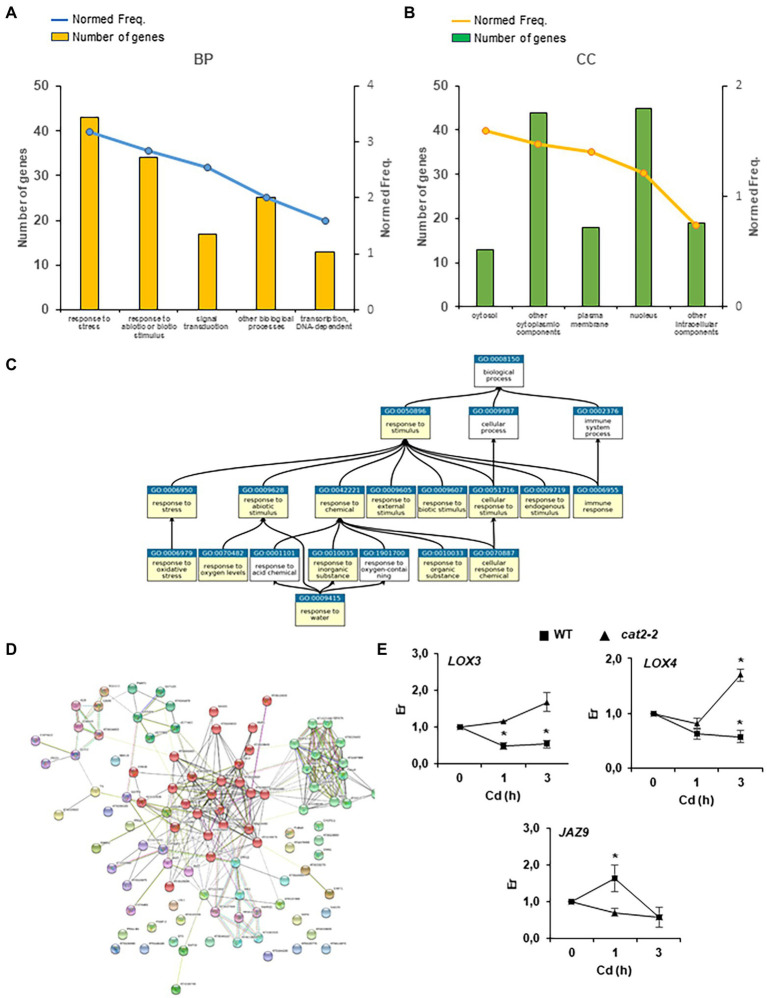
Early peroxisome-dependent genes classification. **(A)** Five main GO categories related to biological processes (BPs) and **(B)** cellular components (CCs) obtained by the Classification Super Viewer tool (http://bar.utoronto.ca/ntools/cgi-bin/). **(C)** The relationship between the selected gene sets Response to Stimulus (GO:0050896) and Response to Chemical Stimulus (GO:0042221) and other significant gene sets in category GO_BP obtained by the PlantGSEA tool (http://systemsbiology.cau.edu.cn/PlantGSEA/). **(D)** StringDB analysis of early peroxisome-dependent genes showed two main clusters: (1) Heat shock transcription factors and proteins (chaperones) in green and (2) Initial signaling cascades with phosphatases 2C, MAPK, ERF transcription factor and JAZ proteins in red. **(E)** JA biosynthetic genes, lipoxygenases 3 and 4 (*LOX3* and *LOX4*) and JA-dependent signaling gene *JAZ9*, expression in WT and *cat2-2* mutants in seedling responses to Cd stress. Relative expression (Er) in control conditions (0 h) was considered to be 1. Values represent means ±SEM. *denotes significant differences between Cd treatment and control in each genotype (*p* < 0,05; *T* student test).

To determine the different biological processes regulated by early peroxisome-dependent transcriptional footprints, we carried out a gene set enrichment analysis of Gene Ontology (GO) and Kyoto Encyclopedia of Genes and Genomes (KEGG) pathways, as well as protein family gene groups using Classification Super Viewer (University of Toronto), StringDB, GeneMania, PlantGSEA and Mapman tools. Several gene groups were significantly overrepresented (*p* < 0.05 and false discovery rate (FDR) < 0.05) among the early peroxisome-dependent genes (Figure 3; Supplementary Figure S1). As expected, the “Response to Stress” and “Response to Stimulus” GO groups were enriched in the different tools used such as Classification Super Viewer, StringDB and PlantGSEA. A representative number of genes and normed frequency obtained by the Classification Super Viewer tool, related to biological processes (BPs) and cellular components (CC), are presented with respect to these GO groups in Figures 3A,B. Furthermore, using the PlantGSEA tool^11^ (Figure 3C), we observed relationships between “Response to Stimulus” (GO:0050896) and “Response to Chemical Stimulus” (GO:0042221) gene sets and other significant gene sets such as “Responses to different stimuli” (abiotic, biotic, endogenous, etc.), “Response to stress” and “Immune response.” Interestingly, almost 45% of the early peroxisome-dependent genes are localized in the nucleus (Figure 3B) according to the cellular component (CC) results obtained by the Classification Super Viewer tool. These results are in accordance with the important GO categories Signal Transduction and Transcription DNA-dependent at the timings used in this study (Figure 3A).

Analysis of Plant Ontology gene sets showed that 90% of the early peroxisome-dependent genes were related to stamen and 80–85% to different reproductive organs such as sepals and flowers (Supplementary Figure S2). Vascular leaf tissue also accounted for 82% of the genes (Supplementary Figure S2). In addition, in the early peroxisome-dependent genes, we found a significant number of genes that are targets of HY5 and AtbHLH (PIF1) transcription factors (TFs; Supplementary Table S6). We used the StringDB tool to organize early peroxisome-dependent genes, resulting into two main clusters: (1) heat shock transcription factors and proteins (chaperones) and (2) initial signaling cascades with phosphatases 2C and MAPK, transcription factor ERF, as well as JA biosynthesis and signaling genes (Figure 3D; Supplementary Table S7). We analyzed genes *LOX3* and *LOX4* involved in JA biosynthesis in plant responses to Cd stress after a short period of treatment in WT and *cat2-2* mutants. We observed a significant repression of these genes in WT plants after Cd treatment, while no repression was observed in *cat2-2* and, unlike in WT, an induction of *LOX4* in the mutants was observed after 3 h treatment (Figure 3E). Under the same conditions, *JAZ9*, involved in JA-dependent signaling, is induced in WT after 1 h treatment, but no significant changes were observed in *cat2-2* mutants (Figure 3E).

Interestingly, GeneMania analysis, with an FDR-adjusted value of p of 0.05, showed that early peroxisome-dependent genes accounted for almost 93% of the co-expression obtained collecting only data with an associated publication. The list of genes was supplemented with a maximum of 20 direct interactors of early peroxisomal-dependent genes (Supplementary Figure S3A). Most of these proteins are HSPs/chaperones, as well as well-known transcription factors related to plant responses to stress such as *ZAT10* and *WRKY 40* (Supplementary Figure S3A).

### Late peroxisome-dependent transcriptional regulation of pathways and gene families

**Figure 4:**
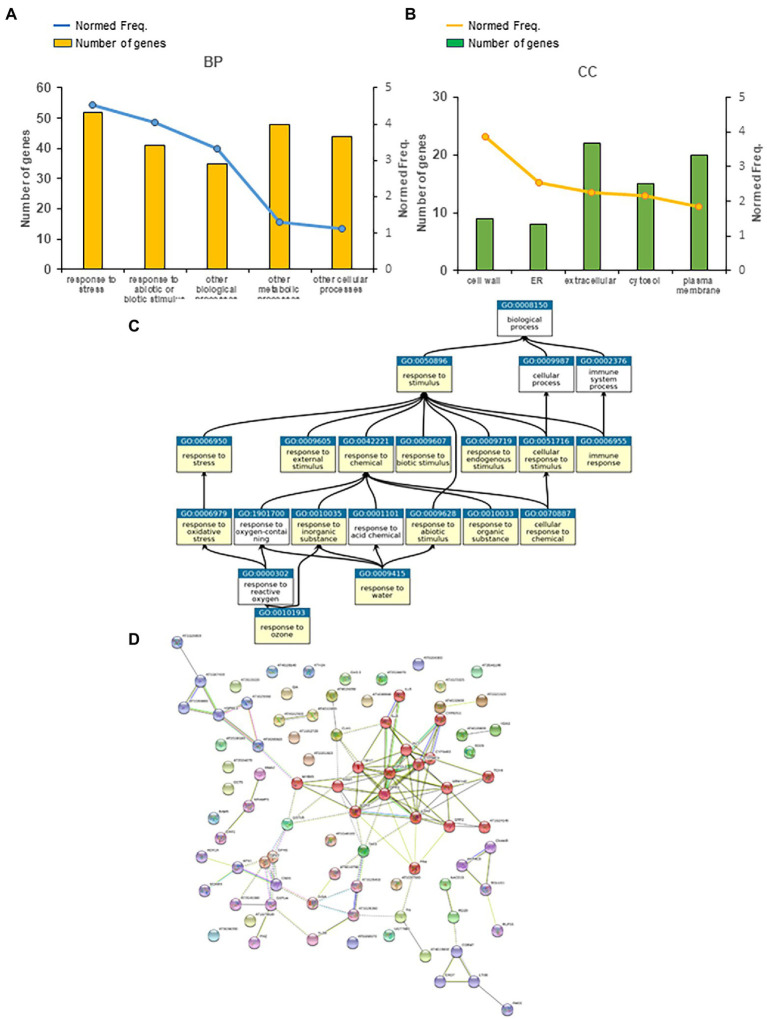
Late peroxisome-dependent genes classification. **(A)** Five main significant GO categories related to biological processes (BPs) and **(B)** cellular components (CCs) obtained using the Classification Super Viewer tool (http://bar.utoronto.ca/ntools/cgi-bin/). **(C)** The relationship between the selected gene sets Response to Stimulus (GO:0050896) and Response to Chemical Stimulus (GO:0042221) and other significant gene sets in category GO_BP obtained using the PlantGSEA tool (http://systemsbiology.cau.edu.cn/PlantGSEA/analysis.php). **(D)** StringDB analysis of late peroxisome-dependent genes showed one main cluster related to JA signaling and alpha-linolenic acid (ALA) metabolism (red), as well as two smaller clusters related to GSH metabolism (medium purple) and heat shock proteins (blue).

We carried out a similar analysis to establish late peroxisome-dependent transcriptional footprints. Several gene groups were significantly overrepresented (*p* < 0.05 and FDR < 0.05) in the late peroxisome-dependent genes (Figure 4; Supplementary Figure S4). A representative number of genes and normed frequencies obtained by the Classification Super Viewer tool, related to biological processes (BP) and cellular components (CC), are presented in Figures 4A,B for these GO groups. Although we found only six genes common to both short-and long-term peroxisome-dependent genes, the “Response to Stress” and “Response to Abiotic and Biotic Stress” GO groups persisted over time (Figures 3A, 4A). The relationships between the gene sets “Response to Stimulus” (GO:0050896) and “Response to Chemical Stimulus” (GO:0042221), as well as other significant gene sets in category GO_BP such as “Responses to abiotic, biotic and endogenous stimuli,” and “Response to stress” and “Immune responses,” identified using the PlantGSEA tool, also persisted^12^ (Figure 4C). During this later period, however, the increase in normed frequency rates is related to cell wall, ER and extracellular locations, with the largest number of genes observed in the extracellular location (Figure 4B) rather than in the nucleus, as observed at the early times (Figure 3B).

Changes in cellular components observed at this later time may be related to the changes observed in biological processes, with a higher normed frequency, other than “Responses to Stress,” found at this timing with respect to early responsive genes, which are “Other Metabolic and Cellular Processes” rather than “Signal Transduction” (Figure 4A). Analysis of Plant Ontology gene sets at this later timing is similar to that carried out at early times, which showed the highest number of genes (almost 90%) in vascular tissue, followed closely by stamen and sepals (Supplementary Figure S5). Interestingly, we also found a significant number of HY5 TF gene targets in late peroxisome-dependent genes (Supplementary Table S6). Analysis using the StringDB tool showed one main cluster for late peroxisome-dependent genes related to JA signaling and alpha-linolenic acid metabolism and two smaller clusters related to GSH metabolism and heat shock proteins (Figure 4D; Supplementary Table S8). Like early peroxisome-dependent genes, late responses accounted for almost 88% of co-expression obtained by GeneMania analysis, supplemented with a maximum of 20 direct interactors of late peroxisomal-dependent genes (Supplementary Figure S3B). In this case, most of these proteins are transcription factors associated with JA signaling such as *TIFY10A (JAZ1)*, *TIFY10B* (*JAZ2*), *TIFY11A* (*JAZ5*), *TIFY5A* (*JAZ8*) and *JAZ10* (Supplementary Figure S3B).

### Peroxisome transcriptional footprints are found in environmental stress-triggered transcriptional responses

**Figure 5:**
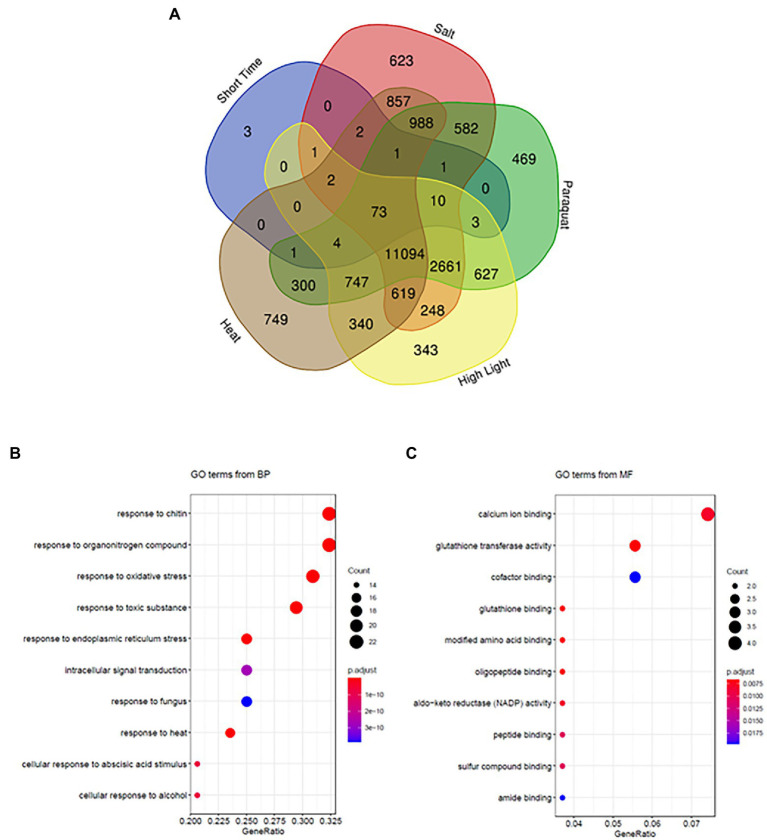
Early peroxisome-dependent genes and abiotic stress responses. **(A)** Comparison using Venny algorithms (http://bioinfogp.cnb.csic.es/tools/venny/) of early peroxisome-dependent genes with transcriptional changes after applying abiotic stress conditions (heat, salt, excess light, and paraquat). **(B)** Enrichment of common genes obtained in **(A)** showing significant GO categories in biological processes (BPs) and in **(C)** molecular function (MF).

We used peroxisome transcriptional footprints to retrieve disturbances through similar transcriptional changes. Different GO groups with the highest normed frequency related to responses to a diverse range of stresses were enriched in peroxisomal transcriptional footprints (Figures 3, 4). We therefore compared early peroxisome-dependent genes with the transcriptional changes observed after applying four representative abiotic stress conditions (heat, salt, excessive light and oxidative stress) caused by the herbicide paraquat (PQ) for a short period of time, as described in a recent analysis (Zandalinas et al., 2021). Interestingly, 72% (73 genes) of the early peroxisomal transcriptional footprints were common to all four stresses (Figure 5; Supplementary Table S9), with 85% (62 genes) being up-regulated in the different stresses (Supplementary Figure S6). Individual comparisons resulted in 83 genes (82.1%) common to heat stress, 90 genes (89.1%) to salt stress and 93 genes (92%) to PQ and high light (Figure 5), and only three genes were not found to be common to any of the stresses compared. Enrichment of the common 73 genes resulted in significant GO_BPs such as responses to chitin, organonitrogen compounds, oxidative stress, toxic substances and to endoplasmic reticulum (ER) stress (Figure 5B). The principal GO processes related to molecular function (MF) are calcium ion binding, GST activity and cofactor binding (Figure 5C). The main KEGG pathways are GSH metabolism and responses to ER stress (Supplementary Table S10).

**Figure 6:**
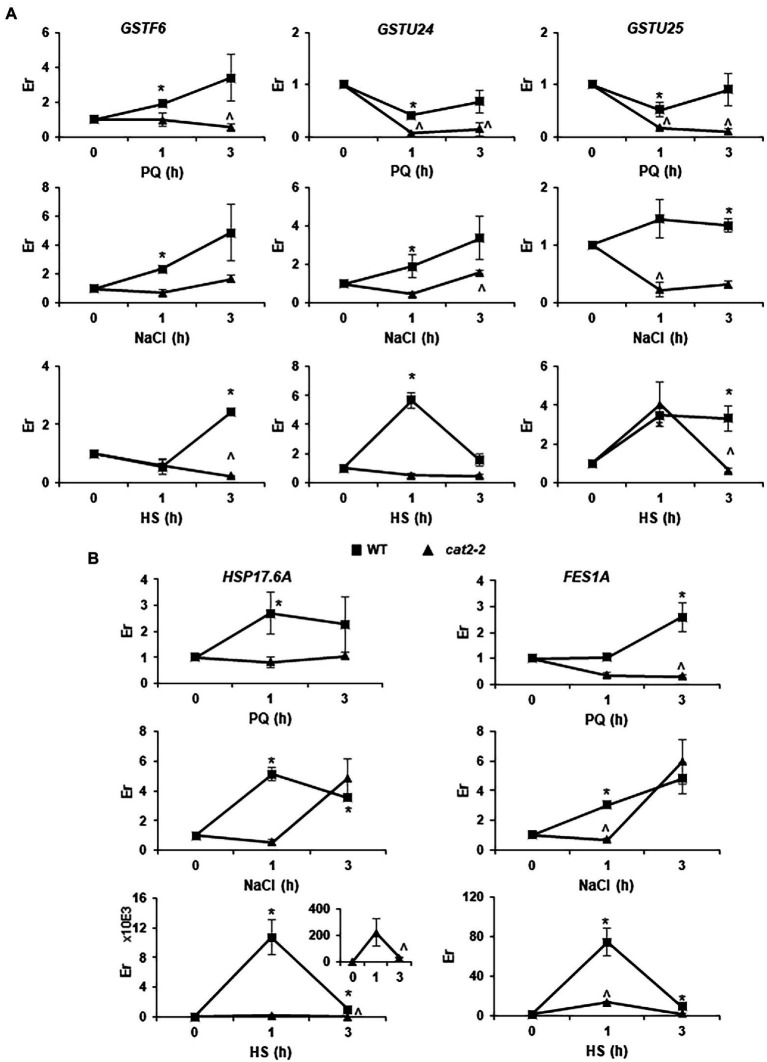
Peroxisomal-dependent gene expression under abiotic stress. GSH metabolism (*GSTF6, GSTU24* and *GSTU25*; **A**) and HSP and ER-stress (*HSP17.6A* and *FES1A*; **B**) related genes were analyzed in WT and *cat2-2* response to paraquat (PQ), salt (NaCl) or heat shock (HS) stress. Relative expression (Er) in control conditions (0 h) was considered to be 1. Values represent means ±SEM. * or ^ denotes significant differences between treatment and control in WT or *cat2-2*, respectively (*p* < 0,05; *T* student test).

To further confirm that peroxisomal-dependent genes highly overlap with genes responsive to different abiotic stresses, we analyzed three genes, *GSTF6*, *GSTU24* and *GSTU25*, involved in GSH metabolism (Supplementary Table S10) and two genes, *HSP17.6A* and *FES1A*, involved in HSP and ER stress pathways (Supplementary Table S7), in plant responses to PQ, salt and HS stress after a short period of treatment in WT and *cat2-2* mutants. We observed a significant induction of *GSTF6* in WT after 1 h of treatment with PQ and salt and, later on, after 3 h under HS conditions (Figure 6A). However, no induction was observed in *cat2-2* mutants, and even a significant repression was observed in these mutants in response to PQ and HS. *GSTU24* and *GSTU25*, which were repressed after 1 h of treatment and recovered their expression after 3 h of treatment in WT, behaved in a very similar way under PQ stress conditions. On the other hand, stronger repression was observed in *cat2-2* mutants, reaching close to zero at 3 h of treatment (Figure 6A). Similarly, an induction of *GSTU24* and *GSTU25* was observed in WT under salt stress, while a milder induction was observed for *GSTU24* after 3 h of treatment and *GSTU25* was repressed in *cat2-2* mutants. Under HS stress, *GSTF6* was induced in WT after 3 h of treatment, while, in *cat2-2* mutants, a repression was observed. *GSTU24* was induced after 1 h of HS stress in WT, while no changes were observed in *cat2-2* mutants. An induction similar to that for *GSTU24* was observed in WT plants under HS stress for *GSTU25*, which was maintained for 3 h, although a repression was observed in *cat2-2* mutants at this time point (Figure 6A). The genes *HSP17.6* and *FES1A* were significantly induced in WT plants following PQ and NaCl treatments, while no changes or repression were observed in *cat2-2* mutants (Figure 6B). Additionally, these genes were observed to be greatly induced under HS in WT plants, although the induction was much lower in *cat2-2* mutants (Figure 6B).

### Relationship of peroxisome transcriptional footprints with ROS and other organelle-dependent transcriptional responses

**Figure 7:**
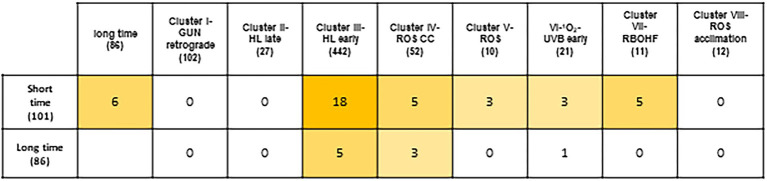
Peroxisome-dependent genes and other ROS footprints. Using the multiple list comparator application (https://www.molbiotools.com/listcompare.php) comparison of early (short-time) and late (long-time) peroxisome-dependent genes with transcriptional changes with different clusters obtained in the ROS wheel (Willems et al., 2016).

Peroxisome transcriptional footprints (PTFs) are based on transcriptomic data from mutant and/or treatments associated with peroxisomal H_2_O_2_ metabolism, and “Response to Oxidative Stress” is one of the GO_BPs found to be significantly overrepresented. We therefore compared PTFs with different transcriptomic data related to ROS. Firstly, we compared early PTFs with the different clusters found in the ROS wheel, resulting from an analysis of 79 microarray studies of redox homeostasis perturbations (Willems et al., 2016). We did not find any common genes in early PTFs with clusters I, II and VIII. Cluster I can be considered a representation of plastid retrograde signaling related to gun mutants (Willems et al., 2016; Figure 7). Cluster II consisted of transcripts triggered by exposure to high light (HL) for 3 and 8 h, which is not the time period used for early PTFs in our analysis. Similarly, cluster VIII consisted of late oxidative stress treatments and constitutively involved redox perturbations. Comparison with the other clusters resulted in 18 genes common to cluster III, related to short exposure to HL, which was included in our analysis of *cat2* mutants. Five genes were found to be common to cluster IV, involving mitochondria and H_2_O_2_ treatment of cell cultures. Three genes were found to be common to cluster V and three genes to cluster VI, involving different ROS inducers and early UV and ^1^O_2_-regulated genes, respectively. Finally, five genes were found to be common to cluster VII, which is related to the impact of RBOHF on a *cat2* background. There were practically no genes common to genes from late PTFs or the different groups from the ROS wheel analysis. We found only five, three and one gene(s) common to clusters III, IV and VI, respectively (Figure 7).

In addition to the comparison of PTFs with group I from the ROS wheel relating to plastids, we compared transcriptomic data from a mutant such as *aox1* altered in mitochondrial ROS metabolism (Giraud et al., 2008) and did not find any gene in common under control or stress conditions.

### Conserved function for plant peroxisome-dependent genes

**Figure 8:**
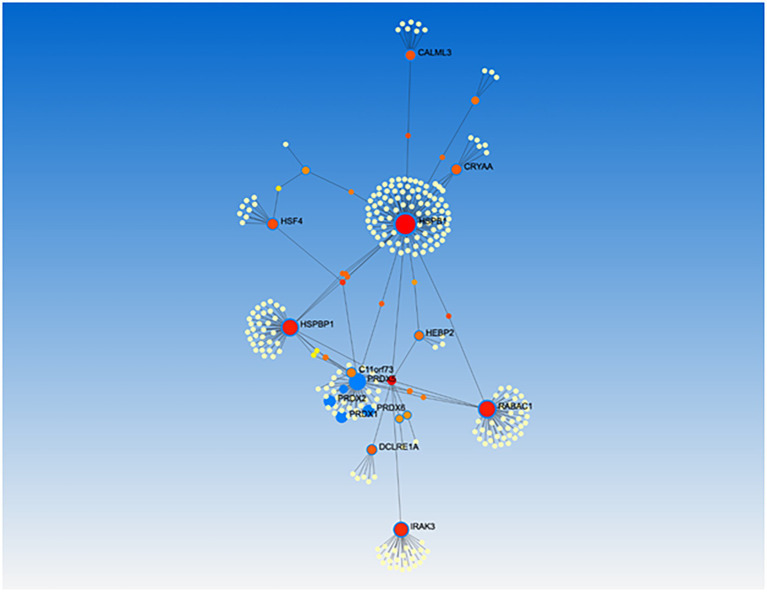
Gene ontology enrichment analysis of human orthologs to Arabidopsis peroxisome-dependent genes. Network analysis were performed using Genemania and NetworkAnalyst 3.0 and resulting networks were submitted to enrichment analysis using GO annotation and pathway databases supported by NetworkAnalyst. Cluster 1 showed peroxidase activity including: PRDX5, PRDX1, PRDX2 and PRDX6, and Cluster 2 showed antioxidant activity, including HSPB1.

Among all *A. thaliana* peroxisome-dependent signaling genes, we managed to assign human orthologs to 21 of their coded proteins (Table 1). Further network analysis using protein–protein interaction data identified a network of 10 clusters relating to these 21 ortholog proteins. Gene ontology enrichment analysis of molecular functions with regard to the 21 orthologs identified a cluster with significant peroxidase activity (cluster 1) which includes PRDX5, PRDX1, PRDX2 and PRDX6, as well as a second cluster with antioxidant activity (Figure 8; Supplementary Table S11). Taking into account only the heat shock proteins, heat shock factors and pPRDX5 from the analysis, we obtained a similar cluster profile, including the antioxidants SOD1 and HSPBP1 in Cluster 1 (Supplementary Figure S7). Interestingly, most of the human proteins identified are directly or indirectly associated with a predisposition to progression or the development of different cancer types (Supplementary Tables S12, S13).

**Table 1:** Domains associated with peroxisome-dependent signaling genes with human orthologs.

Peroxisome-dependent genes with orthologs (*A. thaliana*)	UniProt code (*A. thaliana*)	Protein_alias (*A. thaliana*)	UniProt code (Human)	Protein_alias (Human)
AT1G05680	A0A1W6AJW6	UGT74E1 UDP-glycosyltransferase	A0A140T9Z0	UGT2A1 UDP-glucuronosyltransferase 2A1
AT1G07160	F6LPR6	AP2C2-PP2C-type phosphatase	Q5SGD2	PPM1L-Protein phosphatase 1 l
AT1G22280	F4I1B4	PAPP2C-Phytochrome-associated phosphatase type 2C	Q5SGD2	PPM1L-Protein phosphatase 1 l
AT2G33700	P93006	PP2C27-Probable protein phosphatase 2C 27	Q8N819	PPM1N-Probable phosphatase 1 N
AT1G07400	A0A178WJ08	HSP17.8	A0A140G945	CRYAA-Alpha-crystallin A2
AT1G54050	Q9SYG1	HSP17.4B	P04792	HSPB1-α-crystallin-Hsp20 domain
AT1G78450	Q9SYN6	F3F9.4	Q5THN1	HEBP2-Heme-binding protein 2
AT2G38360	O80915	PRA1B4	Q9UI14	RABAC1-Prenylated Rab acceptor 1
AT3G04000	Q9LX78	F12M12_140-Dehydrogenase-like protein	I6L975	HSDL1-Hydroxysteroid DH like 1
AT3G09350	Q84J81	Fes1A-Hsp70-binding protein	Q9NZL4	HSPBP1 (Fes1A)-Hsp70-binding 1
AT1G33600	Q9FW48	LRR family protein	A6NIV6	LRRIQ4-LRR and IQ domain-containing protein 4
AT1G66080	A0A178W730	nuclear import carrier	Q53FT3	HIKESHI-Protein Hikeshi
AT2G37770	A0A178W1B1	oxidoreductase activity	Q04828	AKR1C1-Aldo-keto reductase family 1 member C1
AT2G45570	F4HRA1	CYP76C5-Cytochrome P450	A0N0X8	CYP1A1-Cytochrome P450
AT3G26680	O64649	Sterile alpha motif (SAM) domain-containing protein	Q6PJP8	DCLRE1A-DNAcross-link repair 1A
AT3G51910	O49402	E2F/DP winged-helixDNA-binding domain	A0A024R6X7	HSF4-HCG2025835, isoform CRA_a
AT4G23190	Q9ZP16	CRK11-Cysteine-rich receptor-like protein kinase 11	Q9Y616	IRAK3-Interleukin-1 receptor-associated kinase 3
AT5G26220	Q8GY54	GGCT2;1-Gamma-glutamylcyclotransferase 2–1	A0A2J8QDS1	CHAC1-γ-glutamylcyclotransferase
AT1G60740	B4G289	PRXIID	P30044	PRDX5
AT2G41100	A0A178VP83	CAM2;CAM3;CAM5	P27482	CALML3-Calmodulin-like protein 3

LRR, Leucine-rich repeat; PRDX5, peroxiredoxin 5; PRXIID, peroxiredoxin.

## Discussion

To properly control organelle function and hence cellular metabolism, different regulatory mechanisms need to exist among the nuclei and organelles to enable information to be mutually exchanged. Retrograde signaling, by which specific signaling molecules used by organelles to transfer information to the nucleus regarding their physiological and developmental state, consequently triggers a regulation of nuclear genes. Retrograde signaling from plastids is best characterized from the initial analysis of GUN proteins, which play a major role in the process, as well as more recent elements such as metabolites and transcription factors (Kleine and Leister, 2016; Wu and Bock, 2021). Mitochondrial retrograde signaling has also been assessed in recent years (Law et al., 2014), although the signal element which transmits information remains unknown; however, multiple upstream regulatory elements, such as TFs from the NAC family, have been identified (De Clercq et al., 2013; Ng et al., 2014). It has been suggested that ROS produced in chloroplasts and mitochondria may play a key role in the retrograde signaling of these organelles (Sun et al., 2011; Huang et al., 2016; Wagner et al., 2018).

Although various analyses of mutants and treatments, leading to altered peroxisomal metabolism, have highlighted specific changes in the transcriptome, little is known about peroxisomal retrograde signaling (Su et al., 2019). Research into wide-scale peroxisome-dependent signaling began with transcriptomic analysis of treatments/mutants that affect catalase, one of the major antioxidants in the organelle, which disturbs peroxisomal H_2_O_2_ metabolism. Thus, treatment with catalase inhibitor 3-aminotriazole (AT) was initially used (Gechev et al., 2005), as well as *cat2* mutants, which affected the principal gene coding for CAT under control and stress conditions (Vanderauwera et al., 2005; Queval et al., 2007, 2012; Chaouch et al., 2010; Mhamdi et al., 2010; Sewelam et al., 2014; Waszczak et al., 2016). In addition, we used transcriptomic analysis of mutants affected in *ACX1* and *GOX2*, which are the principal sources of peroxisomal H_2_O_2_, from β-oxidation and photorespiration pathways, respectively (Sandalio et al., 2021). Double mutants which affect CAT2 and GOX1/2 have also been analyzed (Kerchev et al., 2016). We conducted a meta-analysis of the different transcriptomes available for mutants affected in peroxisomal ROS metabolism, which enabled us to identify a common group of genes that respond to peroxisomal stress. We analyzed early and later time-scale experiments separately given that plants respond at rapid rates (within the seconds-to-minutes time scale) with ROS-dependent signaling pathways which spread signals from stressed tissues throughout the systemic plant-leaves, and also given that different regulators showed a longer time scale and/or waving expression pattern (within hours). Rapid responses lead systemic tissue to become acclimated and prevent related damage, which is vital for improving growth in a changeable environment; later signaling could be involved in acclimation and/or defense responses (Kollist et al., 2019; Zandalinas et al., 2019). Thus, we found 101 and 86 genes that are commonly regulated under short-time and long-time stress treatments, respectively. Enrichment analysis of early peroxisome-dependent genes found GO categories related to responses to stress/stimulus (Figure 3) accordingly to the conditions analyzed. GeneMania analysis showed a high percentage of co-expression in the 101 genes, suggesting an early coordinated peroxisome-dependent plant response to stress. In fact, by comparing these genes with regulated transcripts following different abiotic stresses (Zandalinas et al., 2021), we found that a high percentage of these genes were regulated after all the stresses analyzed (Figure 5). Individual comparisons found that 82.1% of early peroxisome-dependent genes were common to transcriptomic responses to heat stress, with 92% observed to be common to paraquat and high light. Interestingly, 72% of early peroxisome-dependent genes are shared by the four abiotic stresses analyzed, with the associated KEGG pathways related to: (1) GSH metabolism, mainly GST activity, which is associated with detoxification and (2) to the response to ER stress.

In fact, different abiotic stresses can disrupt the correct folding of proteins in the ER, leading to so-called ER stress (Vitale and Boston, 2008; Liu and Howell, 2010). ER stress activates a response system, involving an unfolded protein response (UPR) to re-establish ER homeostasis (Angelos et al., 2017), which involves the induction of different proteins such as molecular chaperones (Angelos et al., 2017). Other ROS, such as the chloroplast singlet oxygen signaling pathway, are involved in ER-dependent protein responses (Beaugelin et al., 2020). Furthermore, the ER membrane-bound NAC domain-containing TF in Arabidopsis ANAC017, which is a master regulator of mitochondria and chloroplast retrograde signaling, has been recently associated with the regulation of the ER UPR (Meng et al., 2019). However, the mechanism underlying peroxisomal H_2_O_2_ dependence and ER stress responses requires further investigation. The peroxisome-ER relationship explored in this study is in line with the evolutionary origin of peroxisomes from the ER in order to deal with a diverse range of pathways involving ROS production, thus preventing oxidative damage to the ER (Gabaldón, 2018).

Further StringDB analysis clustered early peroxisome-dependent genes into two groups related to heat shock factors, chaperones, different TFs, as well as JA biosynthesis and signaling (Figure 3). Previous independent analyses with *cat2-2* mutants have shown that an increase in H_2_O_2_ produced in peroxisomes induces transcripts involved in protein repair responses (Queval et al., 2007; Sewelam et al., 2014); this suggests that peroxisomes could regulate some of the mechanisms leading to protein protection in plant responses to stress. On the other hand, nuclear localization of roughly half of the peroxisome-dependent genes at early time points suggests peroxisome-dependent regulation of transcription. However, most TFs and signaling molecules identified in our meta-analysis were not described by Sewelam and colleagues (Sewelam et al., 2014), probably due to the later timing of the samples analyzed (8 h) in their study. However, a large proportion of differentially regulated TFs were found in double (*cat2/3*) and triple (*cat1/2/3*) catalase mutants involving practically all TF families (Su et al., 2018). Comparison with different transcriptomic data related to ROS showed no common genes with plastid retrograde signaling by GUN proteins (Sewelam et al., 2014) or with mitochondrial *aox1*-dependent signaling (Giraud et al., 2008); these results suggest that ROS signals derived from peroxisomes differ from those from chloroplasts and mitochondria, although interconnections between ROS signals derived from different organelles cannot be ruled out (Sewelam et al., 2014; Su et al., 2018). Thus, in Arabidopsis plants, peroxisomal polyamine-dependent ROS production has been reported to promote the induction of NADPH oxidase activity which, in turn, induces an increase in oxygen consumption by an alternative mitochondrial oxidase pathway (Andronis et al., 2014). Jasmonic acid produced in peroxisomes can also modulate transcriptional and enzymatic changes in plasma membrane NADPH oxidases in rice plants in response to thiocyanate (Yu et al., 2021). The upregulation of *CAT* and *GOX* in Arabidopsis *rboh* mutants in response to Cd (Gupta et al., 2017) points to a close relationship between peroxisomal ROS and NADPH oxidase-dependent ROS production which could play a key role in cellular redox homeostasis and signaling.

Although few peroxisome-dependent genes persist over time, GO processes such as responses to stress and others are maintained, suggesting that one of the main functions of peroxisomal retrograde signaling is to coordinate responses in order to prevent cellular damage and to protect proteins under stress conditions. GeneMania analysis showed a high percentage of co-expression in the 85 genes in late peroxisome-dependent plant responses to stress. Interestingly, in early peroxisome-dependent signaling, the main gene groups are associated with the nucleus, while, with respect to later responses, the main gene groups are associated with the extracellular and plasma membrane. Similar to what occurs at early times, StringDB analysis found a clustered JA-related gene group, whose biosynthesis occurs through peroxisomes, with *ACX1* being one of the primary enzymes involved in JA biosynthesis (Castillo et al., 2004). In turn, JA regulates the number and size of peroxisomes in Arabidopsis plants by repressing *PEX11b* and *PEX11d* (Castillo et al., 2008). Although JA was initially considered a key hormone involved in plant responses to biotic stress, it has now become clear that it is also involved in abiotic stresses such as salinity, wounding, heavy metals and UV (Rodríguez-Serrano et al., 2006; Ghorbel et al., 2021). The regulation of the biosynthesis and signaling events of hormones signaling molecules produced in peroxisomes could be the most natural and efficient way to restore these stress-affected organelles. We found that both JA biosynthesis and JA-dependent signaling are affected in *cat2* mutants in plant responses to Cd stress, thus linking organellar ROS production to JA metabolism. GSH metabolism and heat shock proteins are also two small clustered groups, which points to the role of peroxisome-dependent signaling in cell redox homeostasis and protein protection. HSPs and associated co-chaperones have been reported to be involved in the regulation of JA-dependent responses, thus highlighting the link between both peroxisome-dependent clusters (Di Donato and Geisler, 2019). Recently, Meena et al. (2022) demonstrated that, in wheat, TaHsfA6b-4D, which plays a significant role in linking heat stress responses to unfolded protein responses, is localized in the ER-Golgi complex and peroxisomes under non-stress conditions; however, TaHsfA6b-4D was accumulated in the nucleus following treatment with HS, thus reinforcing the relationship between peroxisomes and ER stress. Furthermore, we showed that all the genes analyzed involved in GSH metabolism or HSP and ER-stress pathways were responsive to different abiotic stresses such as PQ, HS or NaCl in WT plants. These responses were affected in *cat2* mutants linking organellar ROS production to plant responses to abiotic stress related to detoxification, mainly through GST activity and protein repair.

Interestingly, HY5 and PIF1 target genes are overrepresented in early peroxisome-dependent signaling, with HY5 being maintained at a later time. HY5 is a bZIP TF, which directly regulates a wide range of genes mediating plant responses to hormones and abiotic stresses such as cold and UV-B (Ulm et al., 2004; Lau and Deng, 2010; Catalá et al., 2011). PIF1, which belongs to a small family of bHLH TFs that play multiple roles, is mainly accumulated in the dark, induces skotomorphogenesis and facilitates seedling greening processes (Shen et al., 2005; Stephenson et al., 2009). PIF1 regulates the expression of multiple ROS-dependent genes, while PIF1/PIF3 physically interact with HY5/HYH (a HY5 homolog); this gives rise to transcriptional modules that directly bind ROS signaling genes in order to regulate their expression in a coordinated manner (Chen et al., 2013). Different TFs, such as ethylene-responsive transcription factors (ERFs), regulatory proteins (ZAT) and heat shock proteins (HSPs), including HSP17 and HSP90 and MAPKs, are present in ROS-dependent genes regulated by PIF/HY5. Thus, it has been suggested that PIF1/PIF3-HY5/HYH act as a rheostat to fine-tune ROS-dependent signaling pathways (Chen et al., 2013). We found ERF13, ZAT12 and HSP17, among others, to be targets for PIF/HY5 in early peroxisome-dependent genes and for HSP90 in late peroxisome-dependent genes. Previous studies have shown that PIF/HY5 are involved in a peroxisomal dynamic and signaling relationship. Thus, the HYH transcription factor has been linked to peroxisomal proliferation through activation of peroxin 11b (*PEX11b*) via a phytochrome A-dependent pathway (Orth et al., 2007). In addition, PIF1/PIF3-HY5/HYH, a master regulator of plant responses to light conditions, has been shown to regulate the transcription of a number of peroxisomal genes involved in seed development and photosynthesis processes (Kaur et al., 2013). In addition, excessive light induced a phytochrome B-dependent ROS wave involved in rapid stomatal aperture responses, which is essential in plant acclimation to stress (Devireddy et al., 2020).

The ROS gene network originates in the early evolution on Earth and involves virtually all biological systems (Inupakutika et al., 2016). Mittler (2017), in particular, has shown that ROS are crucial for plant and animal life and that several ROS-dependent pathways may be shared. Thus, we identified 21 orthologs for peroxisome-dependent signaling proteins conserved in humans, whose enrichment showed different clusters, related to kinase, antioxidant and peroxidase activities, signaling receptor binding or nuclear receptor activity, among others. Remarkably, most of the 21 orthologs are associated with different stages in the development of cancers (Table 1). This is not surprising given that H_2_O_2_ can promote cell proliferation and differentiation, while CAT overexpression has been shown to reduce the growth of various cell types. Furthermore, low CAT activity has been associated with a higher risk of different cancers, including skin cancer, colorectal cancer, breast cancer and ovarian cancer [reviewed in Lismont et al., 2019]. One of the main proteins in the cluster is HSPBP1, a positive regulator of the proteasomal ubiquitin-dependent protein catabolic process which has been shown to be essential for the development and evolution of cancers such as colon, ovarian, endometrial and prostate cancer (PMID: 25193387, PMID: 21566277, PMID: 12432550). Although its relationship to the development of cancers is complex, HSPBP1 has been shown to be capable of inhibiting apoptosis and of increasing cellular antioxidant capacity (PMID: 21566277). Interestingly, ROS balance may be disrupted by the presence of high levels of iron (Fe^2+^), whose disturbances have been linked to the proliferation of cancer cells. Recently, NEET proteins have been shown to be able to regulate the levels of iron and ROS, thus inhibiting the activation of apoptosis and autophagy in cancer cells (Mittler et al., 2018).

PRXD5, which is found in human peroxisomes and mitochondria (Walbrecq et al., 2015), is a key player in the other cluster identified. The PRXD family of proteins efficiently scavenge peroxides such as hydrogen peroxide, alkyl hydro peroxide, and peroxynitrite and act as sensors and transducers of signaling by H_2_O_2_ (Walbrecq et al., 2015; Rhee, 2016). The induction of PRXD5 has been associated with different cancers, such as ovarian, breast, endometrial, lung cancer, hepatocellular carcinoma and Hodgkin’s lymphoma (Ismail et al., 2019). Its relationship with human orthologs of peroxisome-dependent signaling has not yet been investigated in humans. The PRXD 5-like proteins, AtTPX1 (At1g65980) and AtTPX2 (At1g65970), have been reported in Arabidopsis genome, with thioredoxin-dependent peroxidase activity shown to be present *in vitro* in the protein encoded by AtTPX2 (Verdoucq et al., 1999). Although the peroxisomal location of this protein has not been experimentally demonstrated, human peroxisomes have been clearly shown to act as regulatory hubs in thiol-based signaling networks (Lismont et al., 2019). However, as in the case of plant cells, further research is needed to better understand how cells decode and integrate this peroxisomal ROS-dependent signaling to produce specific responses.

In conclusion, we found that a number of peroxisome-dependent genes are commonly regulated in different mutants and stresses conditions, where peroxisomal ROS metabolism is altered. These highly co-expressed genes are shared with transcriptomic responses to several abiotic stresses. The clustered late peroxisome-dependent gene groups with regard to heat shock factors and proteins, as well as responses to ER stress and GSTs, are mainly involved in protein protection and detoxification. Different transcription factors, in addition to hormone-dependent biosynthesis and signaling, mainly with respect to JA, are present in early peroxisome-dependent genes; this suggests that initial peroxisomal stress may regulate different signaling pathways involved in plant responses to stress. Peroxisome-dependent proteins and human orthologs explored in this study should open up new perspectives and pave the way for further research into how peroxisome-derived H_2_O_2_ in plants and human cells acts as a physiological redox signaling messenger under stress conditions.

## Data availability statement

The names of the repository/repositories and accession number(s) can be found at: https://www.ncbi.nlm.nih.gov/genbank/, GSE199325.

## Author contributions

LT-C searched databases for transcriptomes and performed the meta-analyses. CV designed and supervised the meta-analyses and searched for human orthologs. MP-V performed experiments and made enrichment analyses. AR-G performed experiments and LMS contributed to discussion. MR-P designed the research, supervised the work, and wrote the paper. All authors contributed to the article and approved the submitted version.

## Funding

This study was funded by the Spanish Ministry of Science, Innovation and Universities (MCIU), the State Research Agency (AEI) and FEDER grant PGC2018-098372-B-I00. MP-V was supported by MCIU Research Personnel Training (FPI) grant BES-2016-076518.

## Conflict of interest

The authors declare that the research was conducted in the absence of any commercial or financial relationships that could be construed as a potential conflict of interest.

## Publisher’s note

All claims expressed in this article are solely those of the authors and do not necessarily represent those of their affiliated organizations, or those of the publisher, the editors and the reviewers. Any product that may be evaluated in this article, or claim that may be made by its manufacturer, is not guaranteed or endorsed by the publisher.

## Footnotes

^1^ https://www.ncbi.nlm.nih.gov/geo/

^2^ http://ddlab.sci.univr.it/FunctionalGenomics/

^3^ http://bioinfogp.cnb.csic.es/tools/venny/index.html

^4^ http://bioinformatics.psb.ugent.be/webtools/Venn/

^5^ http://bar.utoronto.ca/ntools/cgi-bin/

^6^ https://string-db.org/

^7^ https://genemania.org/

^8^ https://mapman.gabipd.org/

^9^ https://www.genome.jp/kegg/

^10^ http://systemsbiology.cau.edu.cn/PlantGSEA/analysis.php

^11^ http://systemsbiology.cau.edu.cn/PlantGSEA/

^12^ http://systemsbiology.cau.edu.cn/PlantGSEA/analysis.php
