# Supplementary material for: Gene network downstream plant stress response modulated by peroxisomal H2O2
Source: Front Plant Sci. 2022 Aug 23;13:930721. doi: 10.3389/fpls.2022.930721 (PMC9445673; doi:10.3389/fpls.2022.930721)
Supplement: Supplementary file 1 [file Data_Sheet_1.pdf]

SUPPLEMENTARY MATERIAL

Suppl. Table S1. *Primers used in this work.*

Gene	Primer sequence (5' → 3')	ID
Tubulin beta 4-F	GAGGGAGCCATTGACAACATCTT	AT5G44340
Tubulin beta 4-R	GCGAACAGTTCACAGCTATGTTCA	
Lipoxygenase 3 (LOX3)-F	CACTGCAATTCACAAGCAACC	AT1G17420
Lipoxygenase 3 (LOX3)-R	CAAAGGAGGAATCGGAGAAGC	
Lipoxygenase 4 (LOX4)-F	TGGGTTCTCGTCTAATCTTCGAG	AT1G72520
Lipoxygenase 4 (LOX4)-F	AGGGTTGATGGAGAACTGTGTTC	
JASMONATE-ZIM-DOMAIN PROTEIN 9 (JAZ9)-F	CGTTGCTGCGACTAATGCAA	AT1G70700
JASMONATE-ZIM-DOMAIN PROTEIN 9 (JAZ9)-F	CCAAGAACCGAGCCAAGGAT	
FES1A-F	AAGTAGCAGCATAGATAA	AT3G09350
FES1A-R	GAAGAAGGTTCATTGTAG	
GLUTATHIONE S-TRANSFERASE24 (GSTU24)-F	ATTTCTGGGCGAGTATGTTT	AT1G17170
GLUTATHIONE S-TRANSFERASE24 (GSTU24)-R	TGGATGAGAACCGGAATTTT	
GLUTATHIONE S-TRANSFERASE25 (GSTU25)-F	TGGTTTGAAGCGTATGAGAA	AT1G17180
GLUTATHIONE S-TRANSFERASE25 (GSTU25)-R	CTTAGCTCAGGAACGAACTT	
GLUTATHIONE S-TRANSFERASE 6 (GSTF6)-F	CAGTTGGTTCAAAGCTTGTT	AT1G02930
GLUTATHIONE S-TRANSFERASE 6 (GSTF6)-R	AGGGATAGTGTGAAGATCGA	
HEAT SHOCK PROTEIN 17.6A (HSP17.6A)-F	TCGTAACAACCCTTCAAGAG	AT5G12030
HEAT SHOCK PROTEIN 17.6A (HSP17.6A)-R	CTCCATCCTCACAAACTTCA	

Suppl. Table S2. Works used in the meta-analysis.

Code	Mutant	Tissue	Treatment	Conditions	Time (min)	Reference	Gene selection
**1**	KO *cat2*	adult leave	high light	long day	180	Vanderawera *et al.*, 2005	
**2**	KO *acx1-2*	adult leave	2,4-D	long day	60	Romero-Puertas *et al.*, 2022	
**3**	KO *acx1-2*	adult leave	2,4-D	long day	4320	Romero-Puertas *et al.*, 2022	
**4**	*35S::GOX2*	seedling	Cd (100 µM)	long day	1440	GSE199325	
**5**	*35S::GOX2*	seedling	Cd (100 µM)	long day	30	GSE199325	
**6**	*gox2-1*	seedling	Cd (100 µM)	long day	1440	GSE199325	
**7**	*gox2-1*	seedling	Cd (100 µM)	long day	30	GSE199325	
**8**	KO *cat2*	adult leave	high CO_2 and later normal air	short day	5760	Mhamdi *et al.*, 2010	
**9**	KO *cat2*	adult leave	high CO_2 and later normal air	long day	5760	Mhamdi *et al.*, 2010	
**10**	KO *cat2*	adult leave	high CO_2 and later normal air (short day)	short day	2880	Queval *et al.*, 2012	
**11**	KO *cat2*	adult leave	high CO_2 and later normal air (short day)	short day	5760	Queval *et al.*, 2012	
**12**	KO *cat2*	adult leave	high CO_2 and later normal air (long day)	short day	2880	Queval *et al.*, 2012	
**13**	KO *cat2*	adult leave	high CO_2 and later normal air (long day)	short day	5760	Queval *et al.*, 2012	
**14**	KO *cat2*	adult leave	high CO_2 and later normal air (long day)	long day	480	Sewelam *et al.*, 2014	
**15**	KO *cat2*	adult leave	high light, high CO_2 and later normal air	long day	180	Kerchev *et al.*, 2016	
**16**	*cat2-2gox1-1*	adult leave	high light, high CO_2 and later normal air	long day	180	Kerchev *et al.*, 2016	
**17**	*cat2-2gox2-1*	adult leave	high light, high CO_2 and later normal air	long day	180	Kerchev *et al.*, 2016	
**18**	*cat2 vs. cat2-2gox1-1*	adult leave	high CO_2 and later normal air	long day	180	Kerchev *et al.*, 2016	
**19**	*cat2 vs. cat2-2gox2-1*	adult leave	high CO_2 and later normal air	long day	180	Kerchev *et al.*, 2016	
**20**	KO *cat2*	adult leave	high light, high CO_2 and normal air	long day	1440	Waszczak *et al.*, 2016	
**21**	KO *cat2*	adult leave	high CO_2 and normal air	long day	1440	Waszczak *et al.*, 2016	

SHORT TIME (9 profiles): 1, 2, 5, 7, 15, 16, 17, 18, 19
MEDIUM TIME (1 profile): 14
LONG TIME (11 profiles): 3, 4, 6, 8, 9, 10, 11, 12, 13, 20, 21

Suppl. Table S3. Datasheets used in the meta-analysis

Suppl. Table S4. Early peroxisomal-dependent transcripts.

	AGI ID	Annotation
1	AT1G72680	ATCAD1_CAD1 cinnamyl-alcohol dehydrogenase
2	AT3G51910	AT-HSFA7A_HSFA7A heat shock transcription factor A7A
3	AT2G46240	ATBAG6_BAG6 BCL-2-associated athanogene 6
4	AT4G12400	Hop3 stress-inducible protein, putative
5	AT4G25380	AtSAP10_SAP10 stress-associated protein 10
6	AT3G09350	Fes1A Fes1A
7	AT1G54050	HSP20-like chaperones superfamily protein
8	AT1G28370	ATERF11_ERF11 ERF domain protein 11
9	AT4G38960	BBX19 B-box type zinc finger family protein
10	AT4G24570	DIC2 dicarboxylate carrier 2
11	AT2G41100	ATCAL4_TCH3 Calcium-binding EF hand family protein
12	AT1G28480	GRX480_roxy19 Thioredoxin superfamily protein
13	AT1G19180	AtJAZ1_JAZ1_TIFY10A jasmonate-zim-domain protein 1
14	AT1G02930	ATGST1_ATGSTF3_ATGSTF6_ERD11_GST1_GSTF6 glutathione S-transferase 6
15	AT2G45570	CYP76C2 cytochrome P450, family 76, subfamily C, polypeptide 2
16	AT2G03710	AGL3_SEP4 K-box region and MADS-box transcription factor family protein
17	**AT2G43510**	ATTI1_TI1 trypsin inhibitor protein 1
18	AT1G78410	VQ motif-containing protein
19	AT3G02840	ARM repeat superfamily protein
20	AT4G39670	Glycolipid transfer protein (GLTP) family protein
21	AT5G59820	AtZAT12_RHL41_ZAT12 C2H2-type zinc finger family protein
22	AT5G54490	PBP1 pinoid-binding protein 1
23	AT1G17380	JAZ5_TIFY11A jasmonate-zim-domain protein 5
24	AT2G44840	ATERF13_EREBP_ERF13 ethylene-responsive element binding factor 13
25	AT4G18950	Integrin-linked protein kinase family
26	AT1G57630	Toll-Interleukin-Resistance (TIR) domain family protein
27	AT1G26380	FAD-binding Berberine family protein
28	AT3G03270	Adenine nucleotide alpha hydrolases-like superfamily protein
29	AT1G02450	NIMIN-1_NIMIN1 NIM1-interacting 1
30	AT1G19020	
31	AT4G04490	CRK36 cysteine-rich RLK (RECEPTOR-like protein kinase) 36
32	AT4G02380	AtLEA5_SAG21 senescence-associated gene 21
33	AT1G33600	Leucine-rich repeat (LRR) family protein
34	AT5G13170	AtSWEET15_SAG29_SWEET15 senescence-associated gene 29
35	AT2G26150	ATHSFA2_HSFA2 heat shock transcription factor A2
36	**AT2G37770**	AKR4C9_ChlAKR NAD(P)-linked oxidoreductase superfamily protein
37	**AT2G34600**	JAZ7_TIFY5B jasmonate-zim-domain protein 7
38	AT2G33700	PP2CG1 Protein phosphatase 2C family protein
39	AT1G71000	Chaperone DnaJ-domain superfamily protein
40	AT1G66080	
41	**AT1G07400**	HSP20-like chaperones superfamily protein
42	AT3G16050	A37_ATPDX1.2_PDX1.2 pyridoxine biosynthesis 1.2
43	AT2G47520	AtERF71_ERF71_HRE2 Integrase-type DNA-binding superfamily protein
44	AT4G37990	ATCAD8_CAD-B2_ELI3_ELI3-2 elicitor-activated gene 3-2
45	AT5G57220	CYP81F2 cytochrome P450, family 81, subfamily F, polypeptide 2
46	AT2G32250	FRS2 FAR1-related sequence 2
47	AT1G05680	UGT74E2 Uridine diphosphate glycosyltransferase 74E2
48	AT1G32920	
49	AT1G32540	LOL1 lsd one like 1
50	AT2G39660	BIK1 botrytis-induced kinase1

51	AT5G10695	
52	AT5G12030	AT-HSP17.6A_HSP17.6_HSP17.6A heat shock protein 17.6A
53	AT5G42380	CML37 calmodulin like 37
54	AT3G10300	Calcium-binding EF-hand family protein
55	AT1G22280	PAPP2C phytochrome-associated protein phosphatase type 2C
56	AT4G23190	AT-RLK3_CRK11 cysteine-rich RLK (RECEPTOR-like protein kinase) 11
57	AT3G54420	ATCHITIV_ATEP3_CHIV_EP3 homolog of carrot EP3-3 chitinase
58	AT5G25770	alpha/beta-Hydrolases superfamily protein
59	AT1G60190	AtPUB19_PUB19 ARM repeat superfamily protein
60	AT3G21870	CYCP2;1 cyclin p2;1
61	AT1G28190	
62	AT1G08940	Phosphoglycerate mutase family protein
63	AT1G05560	UGT1_UGT75B1 UDP-glucosyltransferase 75B1
64	AT2G30790	PSBP-2 photosystem II subunit P-2
65	AT1G17170	ATGSTU24_GST_GSTU24 glutathione S-transferase TAU 24
66	AT1G05575	
67	AT5G63450	CYP94B1 cytochrome P450, family 94, subfamily B, polypeptide 1
68	AT5G16970	AER_AT-AER alkenal reductase
69	AT4G01870	tolB protein-related
70	**AT4G15610**	Uncharacterised protein family (UPF0497)
71	AT1G17180	ATGSTU25_GSTU25 glutathione S-transferase TAU 25
72	AT3G26910	hydroxyproline-rich glycoprotein family protein
73	AT2G22200	Integrase-type DNA-binding superfamily protein
74	AT1G22810	Integrase-type DNA-binding superfamily protein
75	AT3G26680	ATSNM1_SNM1 DNA repair metallo-beta-lactamase family protein
76	AT5G65300	
77	AT5G37490	ARM repeat superfamily protein
78	AT1G61610	S-locus lectin protein kinase family protein
79	AT5G15870	glycosyl hydrolase family 81 protein
80	AT5G25450	Cytochrome bd ubiquinol oxidase, 14kDa subunit
81	AT3G04000	NAD(P)-binding Rossmann-fold superfamily protein
82	AT5G39090	HXXXD-type acyl-transferase family protein
83	AT2G38360	PRA1.B4 prenylated RAB acceptor 1.B4
84	AT3G55970	ATJRG21_JRG21 jasmonate-regulated gene 21
85	AT4G15975	RING/U-box superfamily protein
86	AT5G66070	RING/U-box superfamily protein
87	AT5G39050	PMAT1 HXXXD-type acyl-transferase family protein
88	AT5G56100	glycine-rich protein / oleosin
89	AT5G64230	
90	AT5G26220	AtGGCT2;1_GGCT2;1 ChaC-like family protein
91	AT3G23880	F-box and associated interaction domains-containing protein
92	AT2G33830	AtDRM2_DRM2 Dormancy/auxin associated family protein
93	AT3G46110	Domain of unknown function (DUF966)
94	AT3G25610	ATPase E1-E2 type family protein / haloacid dehalogenase-like hydrolase family protein
95	AT5G64310	AGP1_ATAGP1 arabinogalactan protein 1
96	AT5G66650	Protein of unknown function (DUF607)
97	AT1G60740	Thioredoxin superfamily protein
98	AT1G07160	Protein phosphatase 2C family protein
99	**AT1G02850**	BGLU11 beta glucosidase 11
100	AT1G78450	SOUL heme-binding family protein
101	AT3G17611	ATRBL10_ATRBL14_RBL10_RBL14 RHOMBOID-like protein 14

Suppl. Table S5. Late peroxisomal-dependent genes.

	AGI ID	Annotation
1	AT3G44750	ATHD2A_HD2A_HDA3_HDT1 histone deacetylase 3
2	AT1G68765	IDA Putative membrane lipoprotein
3	AT3G48520	CYP94B3 cytochrome P450, family 94, subfamily B, polypeptide 3
4	AT2G38180	SGNH hydrolase-type esterase superfamily protein
5	AT1G52890	ANAC019_NAC019 NAC domain containing protein 19
6	AT1G72520	ATLOX4_LOX4 PLAT/LH2 domain-containing lipoxygenase family protein
7	AT1G07890	APX1_ATAPX01_ATAPX1_CS1_MEE6 ascorbate peroxidase 1
8	AT4G30800	Nucleic acid-binding, OB-fold-like protein
9	AT1G17420	ATLOX3_LOX3 lipoxygenase 3
10	AT2G35980	ATNHL10_NHL10_YLS9__Late embryogenesis abundant (LEA) hydroxyproline-rich glycoprotein family
11	AT3G11340	UGT76B1 UDP-Glycosyltransferase superfamily protein
12	AT5G47220	ATERF-2_ATERF2_ERF2 ethylene responsive element binding factor 2
13	AT4G32800	Integrase-type DNA-binding superfamily protein
14	AT1G74430	ATMYB95_ATMYBCP66_MYB95 myb domain protein 95
15	AT4G28140	Integrase-type DNA-binding superfamily protein
16	AT1G70700	JAZ9_TIFY7 TIFY domain/Divergent CCT motif family protein
17	AT1G20510	OPCL1 OPC-8:0 CoA ligase1
18	AT1G44350	ILL6 IAA-leucine resistant (ILR)-like gene 6
19	AT5G57560	TCH4_XTH22 Xyloglucan endotransglucosylase/hydrolase family protein
20	AT1G79410	AtOCT5_OCT5 organic cation/carnitine transporter5
21	AT5G10760	Eukaryotic aspartyl protease family protein
22	AT1G19670	ATCLH1_ATHCOR1_CLH1_CORI1 chlorophyllase 1
23	AT4G15210	AT-BETA-AMY_ATBETA-AMY_BAM5_BMY1_RAM1 beta-amylase 5
24	AT2G43510	ATTI1_TI1 trypsin inhibitor protein 1
25	AT4G12490	Bifunctional inhibitor/lipid-transfer protein/seed storage 2S albumin superfamily protein
26	AT3G50970	LTI30_XERO2 dehydrin family protein
27	AT3G28210	PMZ_SAP12 zinc finger (AN1-like) family protein
28	AT2G23150	ATNRAMP3_NRAMP3 natural resistance-associated macrophage protein 3
29	AT1G26410	FAD-binding Berberine family protein
30	AT3G04720	AtPR4_HEL_PR-4_PR4 pathogenesis-related 4
31	AT2G38170	ATCAX1_CAX1_RCI4 cation exchanger 1
32	AT1G08830	CSD1 copper/zinc superoxide dismutase 1
33	AT4G31870	ATGPX7_GPX7 glutathione peroxidase 7
34	AT1G51780	ILL5 IAA-leucine resistant (ILR)-like gene 5
35	AT1G80840	ATWRKY40_WRKY40 WRKY DNA-binding protein 40
36	AT2G33380	AtCLO3_CLO-3_CLO3_RD20 Caleosin-related family protein
37	AT2G34600	JAZ7_TIFY5B jasmonate-zim-domain protein 7
38	AT2G27690	CYP94C1 cytochrome P450, family 94, subfamily C, polypeptide 1
39	AT2G24850	TAT_TAT3 tyrosine aminotransferase 3
40	AT2G37770	AKR4C9_ChlAKR NAD(P)-linked oxidoreductase superfamily protein
41	AT2G06050	AtOPR3_DDE1_OPR3 oxophytodienoate-reductase 3
42	AT2G37760	AKR4C8 NAD(P)-linked oxidoreductase superfamily protein
43	AT2G17840	ERD7 Senescence/dehydration-associated protein-related
44	AT3G22370	AOX1A_ATAOX1A_AtHSR3_HSR3 alternative oxidase 1A
45	AT2G37180	PIP2;3_PIP2C_RD28 Aquaporin-like superfamily protein
46	AT5G03630	ATMDAR2 Pyridine nucleotide-disulphide oxidoreductase family protein
47	AT1G20440	AtCOR47_COR47_RD17 cold-regulated 47
48	AT1G07400	HSP20-like chaperones superfamily protein
49	AT3G05660	AtRLP33_RLP33 receptor like protein 33
50	AT1G59860	HSP20-like chaperones superfamily protein
51	AT4G11600	ATGPX6_GPX6_LSC803_PHGPX glutathione peroxidase 6
52	AT5G52640	ATHS83_ATHSP90.1_HSP81-1_HSP81.1_HSP83_HSP90.1 heat shock protein 90.1
53	AT1G73325	Kunitz family trypsin and protease inhibitor protein

54	AT1G26390	FAD-binding Berberine family protein
55	AT4G24350	Phosphorylase superfamily protein
56	AT1G48100	Pectin lyase-like superfamily protein
57	AT3G25180	CYP82G1 cytochrome P450, family 82, subfamily G, polypeptide 1
58	AT3G55920	Cyclophilin-like peptidyl-prolyl cis-trans isomerase family protein
59	AT2G29460	ATGSTU4_GST22_GSTU4 glutathione S-transferase tau 4
60	AT2G41380	S-adenosyl-L-methionine-dependent methyltransferases superfamily protein
61	AT4G15610	Uncharacterised protein family (UPF0497)
62	AT3G14990	AtDJ1A_DJ-1a_DJ1A Class I glutamine amidotransferase-like superfamily protein
63	AT1G76520	PILS3 Auxin efflux carrier family protein
64	AT1G62570	FMO GS-OX4 flavin-monooxygenase glucosinolate S-oxygenase 4
65	AT2G15020	
66	AT2G29450	AT103-1A_ATGSTU1_ATGSTU5_GSTU5 glutathione S-transferase tau 5
67	AT1G24140	Matrixin family protein
68	AT2G04070	MATE efflux family protein
69	AT4G30270	MERI-5_MERI5B_SEN4_XTH24 xyloglucan endotransglucosylase/hydrolase 24
70	AT4G30110	ATHMA2_HMA2 heavy metal atpase 2
71	AT2G41190	Transmembrane amino acid transporter family protein
72	AT3G56200	Transmembrane amino acid transporter family protein
73	AT2G23170	GH3.3 Auxin-responsive GH3 family protein
74	AT1G21550	Calcium-binding EF-hand family protein
75	AT5G54300	Protein of unknown function (DUF761)
76	AT3G44860	FAMT farnesoic acid carboxyl-O-methyltransferase
77	AT1G52720	
78	AT1G65500	
79	AT5G58570	
80	AT2G36970	UDP-Glycosyltransferase superfamily protein
81	AT4G12500	Bifunctional inhibitor/lipid-transfer protein/seed storage 2S albumin superfamily protein
82	AT1G57590	Pectinacetylesterase family protein
83	AT1G02850	BGLU11 beta glucosidase 11
84	AT1G51920	
85	AT1G26800	RING/U-box superfamily protein
86	AT1G21520	

Suppl. Table S6. TFs for early and late peroxisomal-dependent genes.

Early

Gene Set Name (NO. Genes)	Description	Category	NO. Genes in Overlap (k)	p-value	FDR
HY5_CONFIRMED (221)	Confirmed target genes of transcription factor: HY5	TF	8	1.48E-06	4.35E-05
HY5_CONFIRMED_AND_ UNCONFIRMED (260)	Confirmed and Unconfirmed target genes of transcription factor: HY5	TF	8	4.76E-06	6.97E-05
ATBHLH15_CONFIRMED _AND_UNCONFIRMED (749)	Confirmed and Unconfirmed target genes of transcription factor: AtbHLH15	TF	9	1.37E-03	0.0134
ATBHLH15_CONFIRMED (189)	Confirmed target genes of transcription factor: AtbHLH15	TF	4	4.77E-03	0.0349

Late

Gene Set Name (NO. Genes)	Description
HY5_CONFIRMED_AND_UNCONFIRMED (260)	Confirmed and Unconfirmed target genes of transcription factor: HY5
HY5_CONFIRMED (221)	Confirmed target genes of transcription factor: HY5

Suppl. Table S7. StringDB analysis for early peroxisomal-dependent genes.

Cluster color	Gene count	Protein name	Protein identifier
Red	18	CYP94B3	3702.AT3G48520.1
Red	18	JAZ7	3702.AT2G34600.1
Red	18	MYB95	3702.AT1G74430.1
Red	18	CYP94C1	3702.AT2G27690.1
Red	18	ERF2	3702.AT5G47220.1
Red	18	AT1G24140	3702.AT1G24140.1
Red	18	LOX3	3702.AT1G17420.1
Red	18	OPCL1	3702.AT1G20510.1
Red	18	CYP82G1	3702.AT3G25180.1
Red	18	TCH4	3702.AT5G57560.1
Red	18	LOX4	3702.AT1G72520.1
Red	18	TIFY7	3702.AT1G70700.1
Red	18	ILL5	3702.AT1G51780.1
Red	18	WRKY40	3702.AT1G80840.1
Red	18	OPR3	3702.AT2G06050.1
Red	18	FAMT	3702.AT3G44860.1
Red	18	ILL6	3702.AT1G44350.1
Red	18	PR4	3702.AT3G04720.1
Blue	6	AT1G59860	3702.AT1G59860.1
Blue	6	HSP90.1	3702.AT5G52640.1
Blue	6	AT1G07400	3702.AT1G07400.1
Blue	6	AT3G55920	3702.AT3G55920.1

Blue	6	AT1G21550	3702.AT1G21550.1
Blue	6	AT1G26800	3702.AT1G26800.1
Red	26	ERF11	3702.AT1G28370.1
Red	26	AT5G10695	3702.AT5G10695.1
Red	26	AT1G26380	3702.AT1G26380.1
Red	26	AT4G39670	3702.AT4G39670.1
Red	26	AT1G05575	3702.AT1G05575.1
Red	26	AT1G28190	3702.AT1G28190.1
Red	26	AT3G10300	3702.AT3G10300.3
Red	26	CRK36	3702.AT4G04490.1
Red	26	ERF13	3702.AT2G44840.1
Red	26	AT5G66650	3702.AT5G66650.1
Red	26	AT1G32920	3702.AT1G32920.1
Red	26	AT3G25610	3702.AT3G25610.1
Red	26	AT1G19020	3702.AT1G19020.1
Red	26	JAZ1	3702.AT1G19180.1
Red	26	At5g54490	3702.AT5G54490.1
Red	26	AT5G65300	3702.AT5G65300.1
Red	26	AT1G08940	3702.AT1G08940.1
Red	26	RHL41	3702.AT5G59820.1
Red	26	AGP1	3702.AT5G64310.1
Red	26	TCH3	3702.AT2G41100.1
Red	26	AT5G66070	3702.AT5G66070.2
Red	26	AT3G02840	3702.AT3G02840.1
Red	26	CML37	3702.AT5G42380.1
Red	26	SAG21	3702.AT4G02380.1
Red	26	AT1G57630	3702.AT1G57630.1
Red	26	dic-02	3702.AT4G24570.1
Light Green	13	HSFA2	3702.AT2G26150.1
Light Green	13	Hop3	3702.AT4G12400.2
Light Green	13	HSP17.6A	3702.AT5G12030.1
Light Green	13	RBL14	3702.AT3G17611.1
Light Green	13	BAG6	3702.AT2G46240.1
Light Green	13	AT1G07400	3702.AT1G07400.1
Light Green	13	HSFA7A	3702.AT3G51910.1
Light Green	13	PDX1.2	3702.AT3G16050.1
Light Green	13	Fes1A	3702.AT3G09350.1
Light Green	13	AT1G54050	3702.AT1G54050.1
Light Green	13	AT1G66080	3702.AT1G66080.1
Light Green	13	AT5G25450	3702.AT5G25450.1
Light Green	13	AT1G71000	3702.AT1G71000.1

Suppl. Table S8*. StringDB analysis for late peroxisomal-dependent genes.*

**Cluster color**	**Gene count**	**Protein name**	**Protein identifier**
Red	18	CYP94B3	3702.AT3G48520.1
Red	18	JAZ7	3702.AT2G34600.1
Red	18	MYB95	3702.AT1G74430.1
Red	18	CYP94C1	3702.AT2G27690.1
Red	18	ERF2	3702.AT5G47220.1

Red	18	AT1G24140	3702.AT1G24140.1
Red	18	LOX3	3702.AT1G17420.1
Red	18	OPCL1	3702.AT1G20510.1
Red	18	CYP82G1	3702.AT3G25180.1
Red	18	TCH4	3702.AT5G57560.1
Red	18	LOX4	3702.AT1G72520.1
Red	18	TIFY7	3702.AT1G70700.1
Red	18	ILL5	3702.AT1G51780.1
Red	18	WRKY40	3702.AT1G80840.1
Red	18	OPR3	3702.AT2G06050.1
Red	18	FAMT	3702.AT3G44860.1
Red	18	ILL6	3702.AT1G44350.1
Red	18	PR4	3702.AT3G04720.1
Blue	6	AT1G59860	3702.AT1G59860.1
Blue	6	HSP90.1	3702.AT5G52640.1
Blue	6	AT1G07400	3702.AT1G07400.1
Blue	6	AT3G55920	3702.AT3G55920.1
Blue	6	AT1G21550	3702.AT1G21550.1
Blue	6	AT1G26800	3702.AT1G26800.1
Medium Purple	4	APX1	3702.AT1G07890.1
Medium Purple	4	CSD1	3702.AT1G08830.1
Medium Purple	4	MDAR4	3702.AT3G27820.1
Medium Purple	4	AOX1A	3702.AT3G22370.1

Suppl. Table S9. *Common genes obtained in Fig. 5 A*

Suppl. Table S10. *KEGG pathways significantly represented in common genes obtained in Fig. 5 A*

ID	Description	BgRatio	pvalue	p.adjust	geneID
ath00480	Glutathione metabolism	103/5131	0.005148	0.03750	AT1G02930/AT1G17180/AT1G17170
ath04141	Protein processing in ER	215/5131	0.005770	0.03750	AT1G07400/AT5G12030/AT3G09350/AT1G54050

Normed to Freq. in arabidopsis set (± *bootstrap StdDev*, p-value)

Normed freq.	*StdDev*	p-value	Category
Biological process			
3.19	*0.403*	**3.238e-13**	response to stress (Input set freq.: 0.42; 0.13)
2.85	*0.383*	**4.834e-09**	response to abiotic or biotic stimulus (Input set freq.: 0.33; 0.11)
2.54	*0.559*	**2.204e-04**	signal transduction (Input set freq.: 0.16; 0.06)
2.01	*0.316*	**2.488e-04**	other biological processes (Input set freq.: 0.24; 0.12)
1.59	*0.438*	**0.031**	transcription,DNA-dependent (Input set freq.: 0.12; 0.08)
1.31	*0.742*	0.256	electron transport or energy pathways (Input set freq.: 0.01; 0.01)
1.24	*0.121*	**6.673e-03**	other cellular processes (Input set freq.: 0.56; 0.45)
1.13	*0.114*	**0.042**	other metabolic processes (Input set freq.: 0.48; 0.42)
1.12	*0.303*	0.111	developmental processes (Input set freq.: 0.11; 0.1)
0.91	*0.701*	0.369	pollination (Input set freq.: 0; 0.01)
0.83	*0.222*	0.086	protein metabolism (Input set freq.: 0.13; 0.16)
0.76	*0.231*	0.101	cell organization and biogenesis (Input set freq.: 0.07; 0.1)
0.68	*0.243*	0.099	transport (Input set freq.: 0.05; 0.08)
0.47	*0.386*	0.254	DNA or RNA metabolism (Input set freq.: 0; 0.02)
0.38	*0.108*	**1.020e-04**	unknown biological processes (Input set freq.: 0.08; 0.23)
Molecular function			
1.67	*0.574*	**0.039**	transcription factor activity (Input set freq.: 0.09; 0.05)
1.66	*0.273*	**2.238e-03**	protein binding (Input set freq.: 0.26; 0.16)
1.59	*0.193*	**1.714e-03**	other enzyme activity (Input set freq.: 0.31; 0.19)
1.32	*0.203*	**0.014**	other binding (Input set freq.: 0.34; 0.26)
1.3	*0.283*	0.052	transferase activity (Input set freq.: 0.17; 0.13)
1.14	*0.485*	0.156	kinase activity (Input set freq.: 0.05; 0.05)
1.08	*0.277*	0.112	hydrolase activity (Input set freq.: 0.12; 0.11)
1	*0.517*	0.200	nucleic acid binding (Input set freq.: 0.03; 0.03)
0.97	*0.392*	0.180	transporter activity (Input set freq.: 0.04; 0.05)
0.88	*0.304*	0.129	nucleotide binding (Input set freq.: 0.08; 0.1)
0.79	*0.232*	0.082	DNA or RNA binding (Input set freq.: 0.11; 0.14)
0.65	*0.439*	0.222	other molecular functions (Input set freq.: 0.01; 0.03)
0.49	*0.123*	**1.109e-03**	unknown molecular functions (Input set freq.: 0.11; 0.23)
Cellular component			
1.59	*0.387*	**0.031**	cytosol (Input set freq.: 0.12; 0.08)
1.47	*0.159*	**9.560e-04**	other cytoplasmic components (Input set freq.: 0.43; 0.29)
1.4	*0.307*	**0.034**	plasma membrane (Input set freq.: 0.17; 0.12)
1.21	*0.137*	**0.021**	nucleus (Input set freq.: 0.44; 0.36)
1.15	*0.473*	0.171	Golgi apparatus (Input set freq.: 0.04; 0.04)
1.15	*0.239*	0.072	chloroplast (Input set freq.: 0.21; 0.18)
1.09	*0.642*	0.225	cell wall (Input set freq.: 0.02; 0.02)
1.08	*0.493*	0.197	ER (Input set freq.: 0.03; 0.03)
1.05	*0.177*	0.087	other membranes (Input set freq.: 0.25; 0.24)
0.79	*0.193*	0.082	mitochondria (Input set freq.: 0.11; 0.14)
0.74	*0.126*	**0.032**	other intracellular components (Input set freq.: 0.18; 0.25)
0.68	*0.499*	0.341	cytoskeleton (Input set freq.: 0; 0.01)
0.66	*0.292*	0.131	other cellular components (Input set freq.: 0.03; 0.05)
0.6	*0.228*	**0.049**	extracellular (Input set freq.: 0.06; 0.11)
0.17	*0.16*	**0.016**	plastid (Input set freq.: 0; 0.05)
0.14	*0.115*	**6.821e-03**	unknown cellular components (Input set freq.: 0; 0.06)

Absolute values (± *bootstrap StdDev*, p-value)

Absolute value	*StdDev*	p-value	Category
Biological process			
57	*5.4*	**6.673e-03**	other cellular processes (nr. in set: 13044)
49	*4.9*	**0.042**	other metabolic processes (nr. in set: 12333)
43	*5.3*	**3.238e-13**	response to stress (nr. in set: 3826)
34	*4.5*	**4.834e-09**	response to abiotic or biotic stimulus (nr. in set: 3387)
25	*3.8*	**2.488e-04**	other biological processes (nr. in set: 3525)
17	*3.6*	**2.204e-04**	signal transduction (nr. in set: 1901)
14	*3.7*	0.086	protein metabolism (nr. in set: 4771)
13	*3.5*	**0.031**	transcription,DNA-dependent (nr. in set: 2322)
12	*3.1*	0.111	developmental processes (nr. in set: 3034)
9	*2.5*	**1.020e-04**	unknown biological processes (nr. in set: 6705)
8	*2.4*	0.101	cell organization and biogenesis (nr. in set: 2994)
6	*2*	0.099	transport (nr. in set: 2482)
2	*1.1*	0.256	electron transport or energy pathways (nr. in set: 433)
1	*0.8*	0.254	DNA or RNA metabolism (nr. in set: 604)
1	*0.7*	0.369	pollination (nr. in set: 312)
Molecular function			
35	*5.3*	**0.014**	other binding (nr. in set: 7504)
32	*3.8*	**1.714e-03**	other enzyme activity (nr. in set: 5724)
27	*4.3*	**2.238e-03**	protein binding (nr. in set: 4623)
18	*3.8*	0.052	transferase activity (nr. in set: 3923)
13	*3.2*	0.112	hydrolase activity (nr. in set: 3405)
12	*3.4*	0.082	DNA or RNA binding (nr. in set: 4293)
12	*2.9*	**1.109e-03**	unknown molecular functions (nr. in set: 6879)
10	*3.3*	**0.039**	transcription factor activity (nr. in set: 1699)
9	*3*	0.129	nucleotide binding (nr. in set: 2879)
6	*2.5*	0.156	kinase activity (nr. in set: 1486)
5	*1.9*	0.180	transporter activity (nr. in set: 1458)
4	*2*	0.200	nucleic acid binding (nr. in set: 1137)
2	*1.3*	0.222	other molecular functions (nr. in set: 865)
Cellular component			
45	*5*	**0.021**	nucleus (nr. in set: 10525)
44	*4.7*	**9.560e-04**	other cytoplasmic components (nr. in set: 8504)
26	*4.3*	0.087	other membranes (nr. in set: 7037)
22	*4.4*	0.072	chloroplast (nr. in set: 5407)
19	*3.1*	**0.032**	other intracellular components (nr. in set: 7235)
18	*3.8*	**0.034**	plasma membrane (nr. in set: 3639)
13	*3.1*	**0.031**	cytosol (nr. in set: 2321)
12	*2.9*	0.082	mitochondria (nr. in set: 4298)
7	*2.6*	**0.049**	extracellular (nr. in set: 3278)
5	*2*	0.171	Golgi apparatus (nr. in set: 1235)
4	*1.7*	0.131	other cellular components (nr. in set: 1724)
4	*1.8*	0.197	ER (nr. in set: 1051)
3	*1.7*	0.225	cell wall (nr. in set: 780)
1	*0.7*	0.341	cytoskeleton (nr. in set: 414)
1	*0.9*	**0.016**	plastid (nr. in set: 1640)
1	*0.7*	**6.821e-03**	unknown cellular components (nr. in set: 1912)

Suppl. Fig. S1. Enrichment analysis for early peroxisome-dependent genes. Significantly over-represented categories obtained by the Classification Super Viewer (University of Toronto; BAR website: http://bar.utoronto.ca/ntools/cgi-bin/).

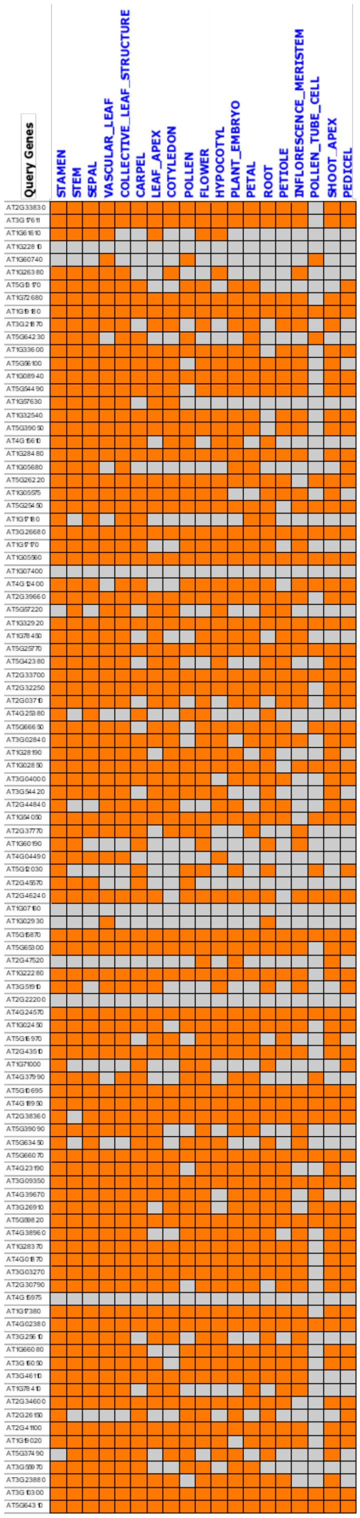

Suppl. Fig. S2. Plant Ontology analysis of early peroxisome-dependent genes.

A)

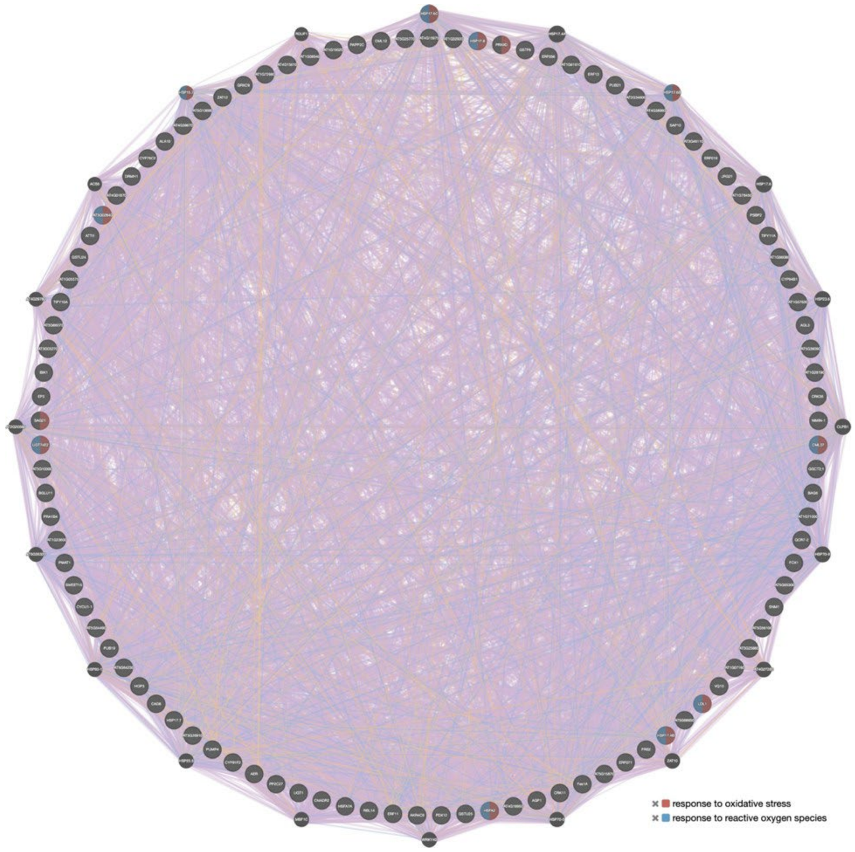

Suppl.Fig.S3.Co-expression analysis of peroxisome-dependent genes. GeneMania analysis showed a high percentage of co-expression in the early peroxisome-dependent genes **(A)** and in late peroxisome-dependent genes **(B)**. Purple Network generated showed co-expression relation between genes. Early peroxisome-dependent genes and late peroxisome-dependent genes, are indicated with stripes which are supplemented with a maximum of 20 direct interactors. Colors show significant enriched terms (FDR < 0.05) related to ROS metabolism.

B)

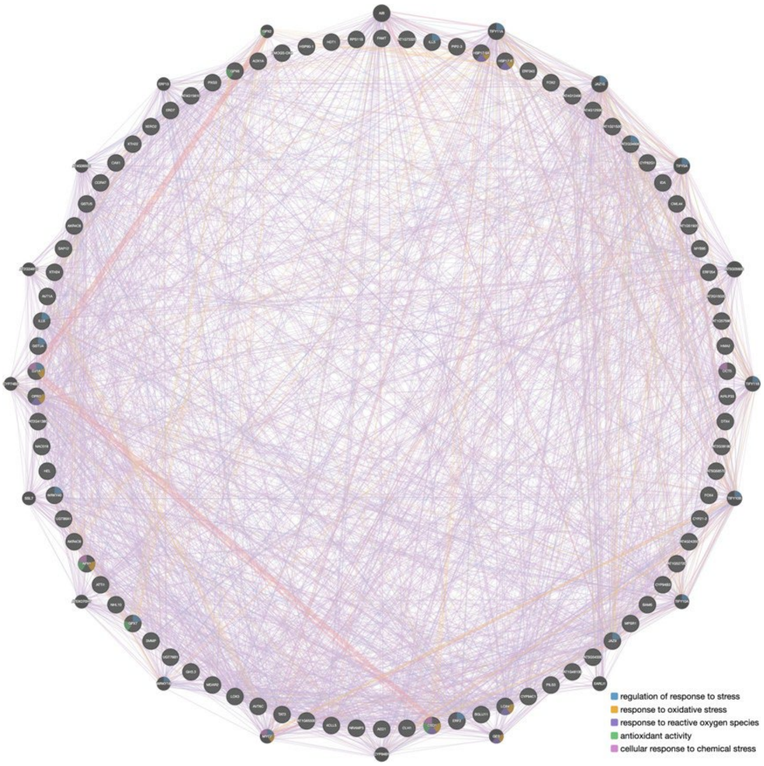

Suppl.Fig.S3.Co-expression analysis of peroxisome-dependent genes. GeneMania analysis showed a high percentage of co-expression in the early peroxisome-dependent genes **(A)** and in late peroxisome-dependent genes **(B)**. Purple Network generated showed co-expression relation between genes. Early peroxisome-dependent genes and late peroxisome-dependent genes, are indicated with stripes which are supplemented with a maximum of 20 direct interactors. Colors show significant enriched terms (FDR < 0.05) related to ROS metabolism.

Normed to Freq. in Arabidopsis set (± *bootstrap StdDev*, p-value)

Biological process			
4.54	*0.406*	**1.79E-24**	response to stress (Input set freq.: 0.6; 0.13)
4.04	*0.464*	**1.54E-16**	response to abiotic or biotic stimulus (Input set freq.: 0.47; 0.11)
3.31	*0.464*	**2.21E-11**	other biological processes (Input set freq.: 0.4; 0.12)
1.58	*0.457*	0.057	signal transduction (Input set freq.: 0.1; 0.06)
1.34	*0.386*	0.085	transport (Input set freq.: 0.11; 0.08)
1.3	*0.107*	**4.79E-03**	other metabolic processes (Input set freq.: 0.55; 0.42)
1.21	*0.328*	0.103	developmental processes (Input set freq.: 0.12; 0.1)
1.12	*0.115*	**0.048**	other cellular processes (Input set freq.: 0.51; 0.45)
1	*0.333*	0.156	transcription,DNA-dependent (Input set freq.: 0.08; 0.08)
0.89	*0.284*	0.138	cell organization and biogenesis (Input set freq.: 0.09; 0.1)
0.77	*0.66*	0.357	electron transport or energy pathways (Input set freq.: 0.01; 0.01)
0.56	*0.174*	**0.022**	protein metabolism(Input set freq.: 0.09; 0.16)
0.34	*0.118*	**1.53E-04**	unknown biological processes (Input set freq.: 0.08; 0.23)
Molecular function			
3.71	*3.216*	0.207	receptor binding or activity (Input set freq.: 0.01; 0)
2.06	*0.679*	**0.018**	transporter activity (Input set freq.: 0.1; 0.05)
1.75	*0.234*	**4.75E-04**	other enzyme activity (Input set freq.: 0.34; 0.19)
1.55	*0.195*	**1.15E-03**	other binding (Input set freq.: 0.4; 0.26)
1.47	*0.374*	**0.036**	hydrolase activity (Input set freq.: 0.17; 0.11)
1.27	*0.332*	0.091	nucleotide binding (Input set freq.: 0.12; 0.1)
1.18	*0.435*	0.153	transcription factor activity (Input set freq.: 0.06; 0.05)
1.17	*0.551*	0.19	nucleic acid binding (Input set freq.: 0.04; 0.03)
1.15	*0.679*	0.221	other molecular functions (Input set freq.: 0.03; 0.03)
1.01	*0.251*	0.116	protein binding (Input set freq.: 0.16; 0.16)
0.85	*0.256*	0.114	transferase activity (Input set freq.: 0.11; 0.13)
0.6	*0.492*	0.316	structural molecule activity (Input set freq.: 0.01; 0.01)
0.54	*0.181*	**0.025**	DNA or RNA binding (Input set freq.: 0.08; 0.14)
0.43	*0.126*	**8.32E-04**	unknown molecular functions (Input set freq.: 0.1; 0.23)
0.22	*0.19*	**0.049**	kinase activity (Input set freq.: 0.01; 0.05)
Cellular component			
3.85	*1.12*	**4.31E-04**	cell wall (Input set freq.: 0.1; 0.02)
2.54	*0.784*	**9.21E-03**	ER (Input set freq.: 0.09; 0.03)
2.24	*0.447*	**1.29E-04**	extracellular (Input set freq.: 0.25; 0.11)
2.15	*0.514*	**2.21E-03**	cytosol (Input set freq.: 0.17; 0.08)
1.83	*0.364*	**2.67E-03**	plasma membrane (Input set freq.: 0.23; 0.12)
1.62	*0.653*	0.088	Golgi apparatus (Input set freq.: 0.06; 0.04)
1.6	*0.241*	**3.63E-03**	chloroplast (Input set freq.: 0.3; 0.18)
1.55	*0.488*	0.071	other cellular components (Input set freq.: 0.09; 0.05)
1.41	*0.168*	**4.68E-03**	other cytoplasmic components (Input set freq.: 0.41; 0.29)
1.24	*0.196*	**0.04**	other intracellular components (Input set freq.: 0.31; 0.25)
1.18	*0.213*	0.059	other membranes (Input set freq.: 0.29; 0.24)
1.01	*0.435*	0.181	plastid (Input set freq.: 0.05; 0.05)
0.63	*0.519*	0.328	ribosome (Input set freq.: 0.01; 0.01)
0.6	*0.108*	**1.49E-03**	nucleus (Input set freq.: 0.22; 0.36)
0.46	*0.198*	**0.012**	mitochondria (Input set freq.: 0.06; 0.14)
0.17	*0.133*	**0.016**	unknown cellular components (Input set freq.: 0.01; 0.06)

Absolute values (± *bootstrap StdDev*, p-value)

Biological process			
52	*4.6*	**1.79E-24**	response to stress (nr. in set: 3826)
48	*3.8*	**4.79E-03**	other metabolic processes (nr. in set: 12333)
44	*4.4*	**0.048**	other cellular processes (nr. in set: 13044)
41	*4.6*	**1.54E-16**	response to abiotic or biotic stimulus (nr. in set: 3387)
35	*4.8*	**2.21E-11**	other biological processes (nr. in set: 3525)
11	*2.9*	0.103	developmental processes (nr. in set: 3034)
10	*2.8*	0.085	transport (nr. in set: 2482)
9	*2.5*	0.057	signal transduction (nr. in set: 1901)
8	*2.5*	0.138	cell organization and biogenesis (nr. in set: 2994)
8	*2.4*	**0.022**	protein metabolism(nr. in set: 4771)
7	*2.3*	**1.53E-04**	unknown biological processes (nr. in set: 6705)
7	*2.2*	0.156	transcription,DNA-dependent (nr. in set: 2322)
1	*0.8*	0.357	electron transport or energy pathways (nr. in set: 433)
Molecular function			
35	*4.3*	**1.15E-03**	other binding (nr. in set: 7504)
30	*3.9*	**4.75E-04**	other enzyme activity (nr. in set: 5724)
15	*3.7*	**0.036**	hydrolase activity (nr. in set: 3405)
14	*3.4*	0.116	protein binding (nr. in set: 4623)
11	*2.8*	0.091	nucleotide binding (nr. in set: 2879)
10	*2.9*	0.114	transferase activity (nr. in set: 3923)
9	*2.5*	**8.32E-04**	unknown molecular functions (nr. in set: 6879)
9	*2.9*	**0.018**	transporter activity (nr. in set: 1458)
7	*2.3*	**0.025**	DNA or RNA binding (nr. in set: 4293)
6	*2.1*	0.153	transcription factor activity (nr. in set: 1699)
4	*1.8*	0.19	nucleic acid binding (nr. in set: 1137)
3	*1.7*	0.221	other molecular functions (nr. in set: 865)
1	*0.8*	0.316	structural molecule activity (nr. in set: 556)
1	*0.8*	0.207	receptor binding or activity (nr. in set: 90)
1	*0.8*	**0.049**	kinase activity (nr. in set: 1486)
Cellular component			
36	*4.2*	**4.68E-03**	other cytoplasmic components (nr. in set: 8504)
27	*4.2*	**0.04**	other intracellular components (nr. in set: 7235)
26	*3.8*	**3.63E-03**	chloroplast (nr. in set: 5407)
25	*4.4*	0.059	other membranes (nr. in set: 7037)
22	*4.3*	**1.29E-04**	extracellular (nr. in set: 3278)
20	*3.9*	**2.67E-03**	plasma membrane (nr. in set: 3639)
19	*3.3*	**1.49E-03**	nucleus (nr. in set: 10525)
15	*3.5*	**2.21E-03**	cytosol (nr. in set: 2321)
9	*2.5*	**4.31E-04**	cell wall (nr. in set: 780)
8	*2.4*	**9.21E-03**	ER (nr. in set: 1051)
8	*2.4*	0.071	other cellular components (nr. in set: 1724)
6	*2.5*	**0.012**	mitochondria (nr. in set: 4298)
6	*2.3*	0.088	Golgi apparatus (nr. in set: 1235)
5	*2.1*	0.181	plastid (nr. in set: 1640)
1	*0.7*	**0.016**	unknown cellular components (nr. in set: 1912)
1	*0.8*	0.328	ribosome (nr. in set: 525)

Suppl. Fig. S4. Enrichment analysis for late peroxisome-dependent genes. Significantly over-represented categories obtained by the Classification Super Viewer (University of Toronto; BAR website: http://bar.utoronto.ca/ntools/cgi-bin/).

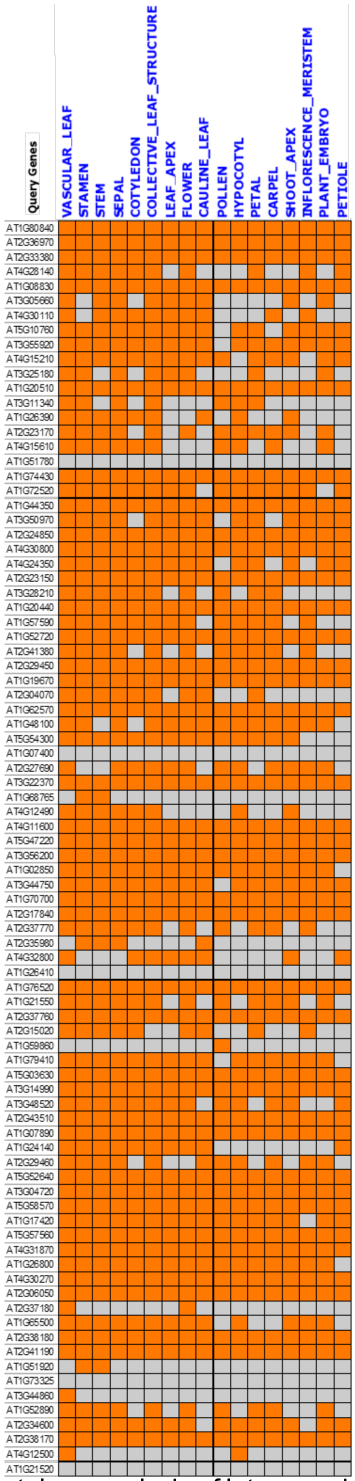

Suppl. Fig. S5. Plant Ontology analysis of late peroxisome-dependent genes.

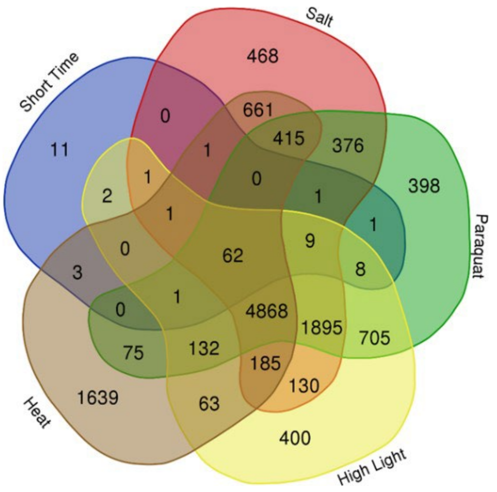

Suppl. Fig. S6: Comparison of early peroxisome transcriptional footprints and upregulated genes in response to stress. Comparison using Venny algorithms (http://bioinfogp.cnb.csic.es/tools/venny/) of early peroxisome-dependent genes with transcriptional changes after applying abiotic stress conditions considering up-regulated genes (heat, salt, excess light, and paraquat).

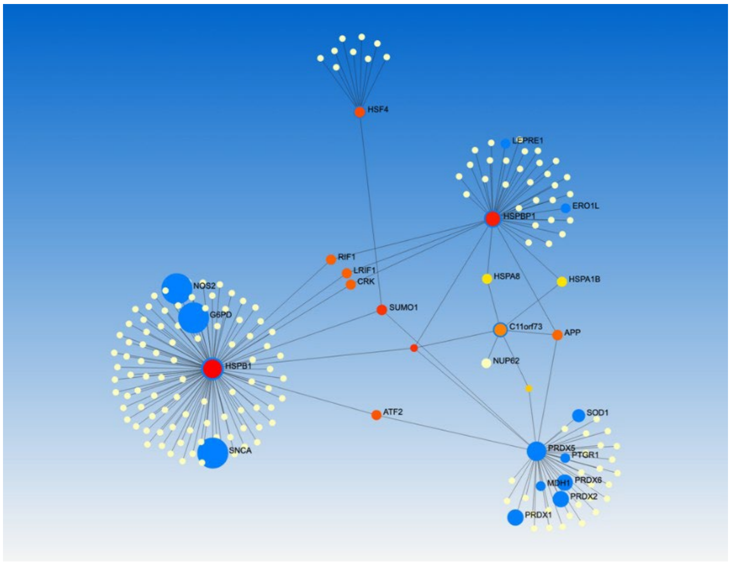

Suppl. Fig. S7: Gene ontology enrichment analysis of interesting human orthologs to Arabidopsis peroxisome-dependent genes. Network analysis with HSPBP1, HSF4, HSPB1, HIKESHI protein and PRXD5. Cluster 1 showed peroxidase activity including: PRDX5, PRDX1, PRDX2 and PRDX6, and Cluser 2 two showed antioxidant activity, including SOD1 and HSPB1.
